# Beyond the mean: genetic control of gene expression fidelity and dispersion

**DOI:** 10.64898/2026.04.01.715853

**Published:** 2026-04-07

**Authors:** Brendan Jamison, Alexander Chen, Erik McIntire, Xin He, Yoav Gilad

**Affiliations:** 1 Department of Human Genetics, The University of Chicago, Chicago, IL 60637, USA; 2 Department of Medicine, Section of Genetic Medicine, The University of Chicago, Chicago, IL 60637, USA

## Abstract

For decades, molecular biologists have interpreted gene regulation through measurements of mean gene expression, because they could not resolve regulatory variation among individual cells. The advent of single-cell genomics has now made that variation measurable, revealing pervasive differences in gene expression among apparently similar cells. Whether this variation mainly reflects stochastic noise or an informative regulatory property remains unclear. Here we show that mean-corrected gene expression dispersion is a reproducible and biologically structured feature of gene regulation that reflects regulatory fidelity. In heterogeneous differentiated cardiac cultures, genes with low dispersion are shared across cell types, enriched for housekeeping functions, depleted for expression quantitative trait loci, and more highly connected in transcriptional and protein interaction networks. In a comparative single-cell system spanning human, chimpanzee, and allotetraploid cells, a substantial subset of interspecies differences in regulatory dispersion persists in a shared *trans* environment, indicating that gene expression fidelity is often regulated in *cis*. Our findings establish gene expression dispersion as a genetically encoded dimension of gene regulation that is distinct from mean expression, and places dispersion along a fidelity-plasticity axis with implications for development, disease, and threshold-dependent cellular phenotypes.

## Introduction

Gene expression studies have always sought to explain how biological systems differ from one another, between tissues, across conditions, and among individuals^1–4^. During most of this time, expression measurements were obtained from bulk samples. Every estimate of gene expression, therefore, was necessarily an average across many individual cells, and the only relevant quantity that could be compared across samples was the mean. For decades, our research and our understanding of molecular biology, genetics, and genomics have been built on comparisons of mean expression levels, treating the mean as sufficiently informative to model biology.

The emergence of single-cell transcriptomics changed the scale of measurement, allowing researchers to quantify gene expression in individual cells^5^. This change in measurement resolution enabled a wave of studies focused on the mechanistic basis of transcription itself, including the characterization of transcriptional bursting, stochastic promoter switching, and the dynamics of RNA production and degradation at single-cell resolution^6,7^. These studies established that gene expression is inherently dynamic and stochastic at the molecular level, and that cell-to-cell variability can arise directly from fundamental properties of transcriptional regulation^8^.

Yet, despite this technological advance and the new scale of measurement, most analyses aimed at comparing gene expression between systems have continued to rely on the same mean-based conceptual framework. Even with single-cell data, the standard approach is to aggregate measurements across what are presumed to be homogeneous groups of cells, producing a “pseudobulk” profile that again represents the average expression across many cells^9–11^. In that sense, the advantage of single-cell data largely became the ability to better identify homogeneous groups of cells^12^, enabling comparisons between more precisely defined cell populations. But even at this higher resolution, the analysis still relied on means, derived from pseudobulk profiles rather than from the full distribution of single-cell gene expression levels^13–15^.

At the same time, when we measure gene expression at single-cell resolution, we find that even among apparently homogeneous cell populations, individual cells do not all express each gene at the same level^16–18^. This variability, which we refer to as dispersion of gene expression, has been studied in certain biological contexts. During development and differentiation, for example, changes in cell-to-cell variability have been shown to accompany fate transitions^19,20^. At the mechanistic level, dispersion of gene expression has been linked to transcriptional bursting and RNA turnover dynamics^21–23^.

Still, most analyses aimed at comparing gene expression across biological systems continue to treat cell-to-cell variability primarily as technical or stochastic noise. Consequently, the rich literature on transcriptional dynamics has not been fully integrated into how gene expression is analyzed as a phenotype. In large part, this disconnect reflects the fact that dispersion has been primarily interpreted through a mechanistic lens, as an emergent consequence of transcriptional dynamics such as promoter-specific bursting behavior, rather than being examined as a property of regulatory systems with potential functional significance.

Indeed, most prior studies that considered the functional consequences of regulatory dispersion have examined differences between individuals rather than between individual cells^24,25^. For example, after an immune challenge, the variance in gene expression among individuals often decreases even when the mean across the population does not change, because all individuals mount a similar response^26,27^. Such analyses describe how the distribution of mean expression shifts among people or conditions, but they do not reveal how expression varies among cells within a single sample. In parallel, studies that directly examined cell-to-cell variability have primarily focused on the mechanistic origins of dispersion, including properties of transcriptional machinery^28,29^, promoter architecture^30^, enhancer regulation^31^, and RNA kinetic processes^32^. While these studies have provided important insight into how dispersion arises, they have not explicitly examined regulatory dispersion as a functional property of gene regulation.

The role and functional importance of expression dispersion thus remain unclear. Understanding whether dispersion of gene expression represents a biological signal is a fundamental question, because the implications extend to many aspects of biology that are governed by threshold effects. Many genetic and physiological processes operate near thresholds, where a shift in expression can move some cells beyond a critical boundary while others remain below it^33,34^. Traditionally, such threshold-dependent outcomes have been interpreted as consequences of changes in mean expression between individuals or conditions. However, differences in expression dispersion could produce similar effects. Distinguishing whether threshold effects arise from differences in mean or differences in dispersion will fundamentally change how we interpret the biology of thresholds and how we think about mechanisms of variation in health and disease. Although our study focuses specifically on determining whether dispersion is biological rather than technical, the answer to this question carries broad implications for understanding and eventually manipulating biological variability.

## Results

### Estimating mean-corrected dispersion in single-cell gene expression data

**Figure 1.**
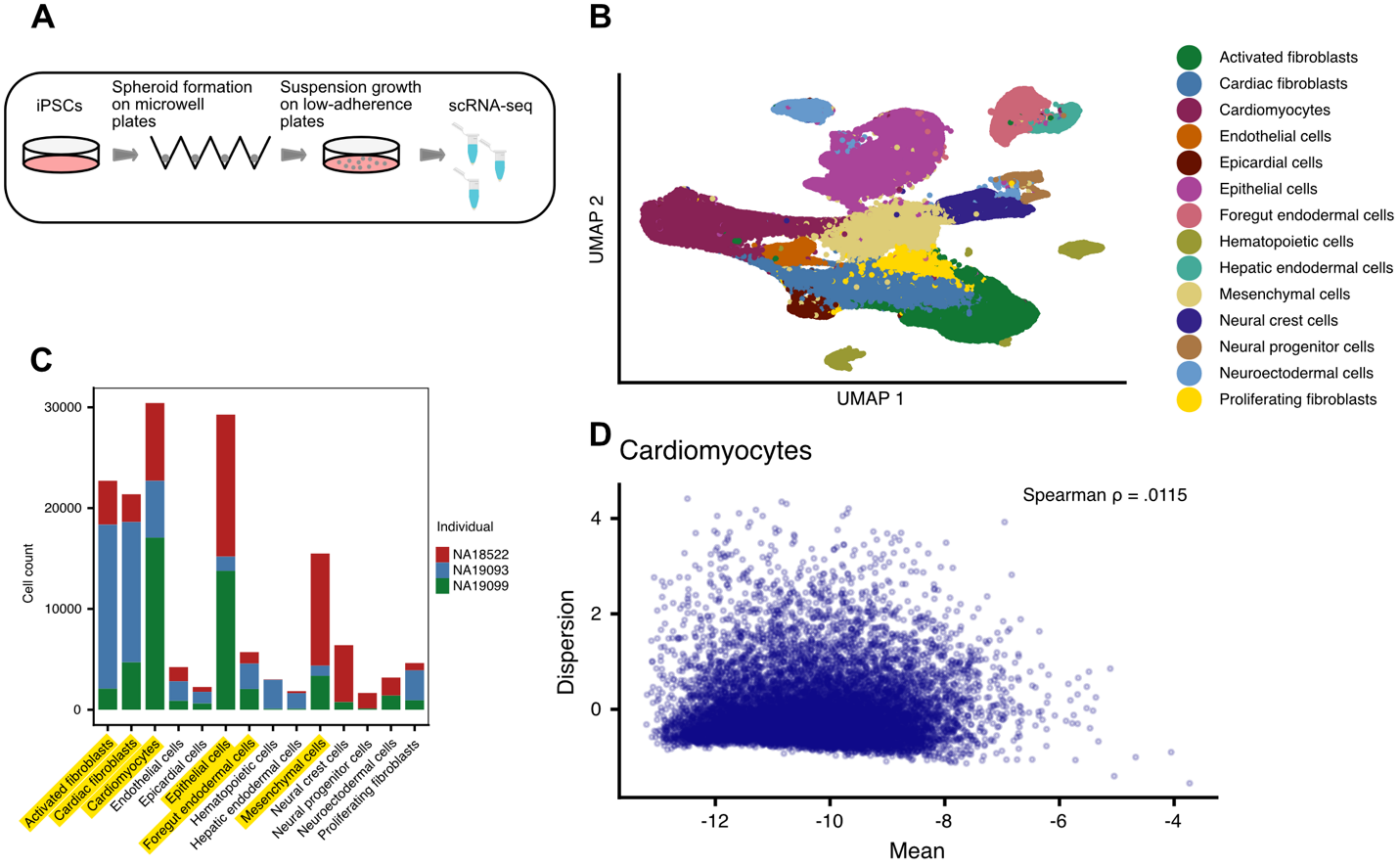
Experimental model and mean-independent dispersion. **A.** Overview of experimental procedure. iPSCs were aggregated into spheroids on microwell plates. After one day, cardiac HDCs were transferred to low-adherence plates and cultured for 12 days. **B.** UMAP projection of all single cells after quality control filtering and reference map-based annotation. **C.** The number of cells per cell type for each individual. Cell types above the threshold of 1000 cells per individual are highlighted in yellow. **D.** Representative sample demonstrating the independence of dispersion from mean expression (ρ = 0.0115). Dispersion and expression estimates are natural log transformed.

We characterized gene expression dispersion in single cell data from cardiac heterogeneous differentiated cultures (cardiac HDCs^35^) of three unrelated individuals. The cardiac HDCs were generated in a single batch and maintained for 14 days. Following differentiation, cells from the three individuals were dissociated and combined in equal proportions prior to single-cell RNA sequencing (Figure 1A). After genotype-based demultiplexing and quality control filtering (Methods; Supplementary Figure S1), we retained data from 152,223 high-quality cells, with a mean sequencing depth of 87,329 reads per cell.

Cell type annotation was performed using a label-transfer approach based on a previously characterized cardiac HDC reference dataset containing 14 annotated cell types^35^. Across the entire dataset, 96% of cells were labeled (confidence score greater than 0.8)^36^, and we detected all 14 expected cell types (Figure 1B). To have adequate statistical power for estimating gene expression dispersion, we restricted downstream analyses to the six cell types represented by at least 1,000 cells in each individual (Figure 1C).

We used Memento^37^ to estimate gene expression dispersion in each combination of individual and cell type (3 individuals, 6 cell types, for a total of 18 samples). Memento adjusts for the dependence of variance on mean expression and produces a residual variance metric. Henceforth we refer to this mean-adjusted residual variance as dispersion. Across the 18 samples, we estimated dispersion for 11,847 genes with a mean raw UMI of 0.07 or above (the default UMI threshold set by the authors of Memento). We confirmed that the dispersion is largely independent of the mean expression (Figure 1D; Supplementary Figures S2–S3; Supplementary Table S1).

### Dispersion exhibits structured patterns across cell types

If dispersion of single cell gene expression is a structured regulated process, genes that perform general cellular functions rather than cell type–specific roles may exhibit similar levels of dispersion across cell types. To examine this, we ranked genes by their median dispersion (across individuals) within each cell type and compared rankings across cell types. We identified 1,035 genes that consistently fell within the lowest quintile of dispersion across all six cell types. Under a model in which dispersion rankings are independent across cell types, fewer than one gene would be expected to overlap across all six.

**Figure 2.**
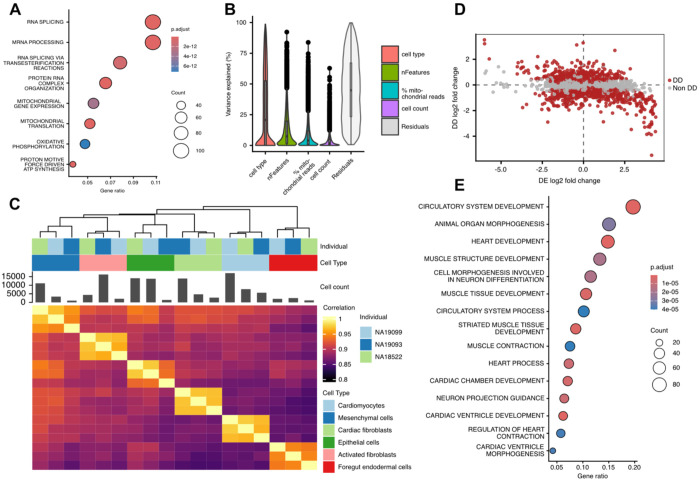
Dispersion patterns are structured across cell types. **A.** Gene Ontology (GO) enrichment analysis for genes ranked in the lowest quintile of dispersion in every cell type. **B.** Variance partition analysis showing the percent of variance in dispersion estimates across samples that is explained by biological and technical variables. **C.** Correlation structure of dispersion profiles across samples. **D.** Relationship between the differential dispersion (DD) and differential mean expression (DE) effect sizes for each gene in the differential tests between cardiomyocytes and foregut endodermal cells. Genes with significant differential dispersion (FDR < 0.05) are colored in red. **E.** GO enrichment analysis of genes that are high DE and low DD in cardiomyocytes relative to foregut endodermal cells.

We found that shared low-dispersion genes tend to have relatively high mean expression levels, possibly consistent with their broad functional importance (this observation is not explained by an overall correlation between dispersion and mean, which are uncorrelated; Methods; Supplementary Figure S4). Gene ontology enrichment analysis of the shared low-dispersion gene set revealed strong enrichment for housekeeping processes, including mRNA processing, ribonucleoprotein complex biogenesis, and ATP biosynthetic process (*P* < 10^−10^; Methods; Figure 2A). These functional enrichments were robust to alternative clustering resolutions of the data (Supplementary Figure S5; Supplementary Table S2).

We next focused on genes whose expression dispersion differs across cell types. Using variance partition analysis (Methods), we found that cell type accounts for the largest proportion of variance (median of 20.7%) in dispersion estimates in our data (Figure 2B). Clustering based on dispersion estimates yields grouping by cell type, similar to clustering based on mean expression levels, though dispersion and mean expression themselves are uncorrelated (Figure 2C; Supplementary Figures S2–3 & S6).

We identified genes with different expression dispersion across cell types (we refer to these as Differentially Dispersed or DD genes) by fitting a linear model with cell type as a fixed effect (Methods). We used permutation of cell type labels to obtain a null distribution, calculate empirical *P* values, and estimate false discovery rate (FDR; Supplementary Figure S7). At an empirical FDR < 0.05, we identified 1,065 to 3,722 DD genes between pairs of cell types (Supplementary Table S3). To further examine the relationship between mean expression levels and dispersion, we also fitted an analogous linear model to mean expression estimates, identifying 2,585 to 6,924 differentially expressed (DE) genes between cell types (Supplementary Table S3).

We examined the DD genes between cell types. We focused on the subset of DE/DD genes that show high expression but low dispersion in one cell type compared to the other. This pattern may indicate that, in a particular cell type, these highly expressed genes need to be regulated with high fidelity. Indeed, we found that such genes are enriched with cell-type specific functions (Supplementary Table S2). For example, in the pairwise comparison between cardiomyocytes and foregut endodermal cells, genes with higher mean expression and lower dispersion in cardiomyocytes were enriched for heart development, striated muscle tissue development, cardiac chamber morphogenesis, and cardiac ventricle development (*P* < 10^−5^; Methods; Figure 2D-E).

### Dispersion is associated with regulatory fidelity and functional pleiotropy

Having established that dispersion of single cell gene expression is structured, we next examined its relationship to other properties of gene regulation to assess whether it reflects regulatory fidelity. We first considered the relationship of dispersion with expression quantitative trait loci (eQTLs). If low dispersion reflects high regulatory fidelity, such genes may be less tolerant of regulatory mutations and therefore less likely to be associated with eQTLs.

**Figure 3.**
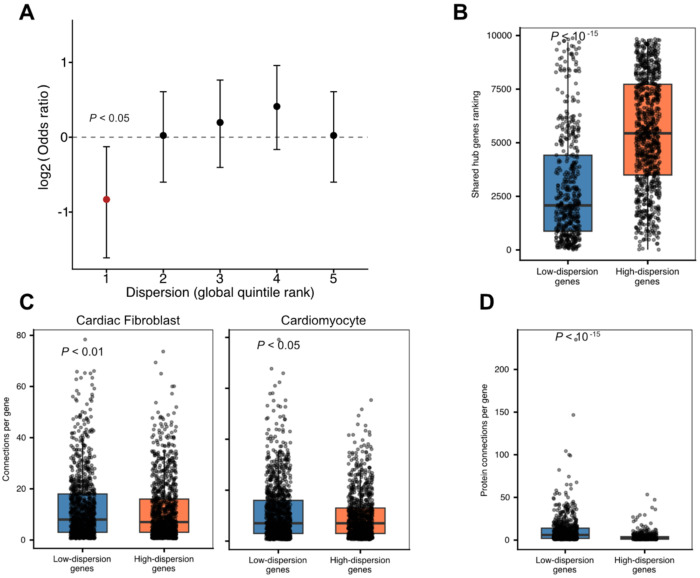
Low dispersion is associated with greater regulatory constraint and pleiotropy. **A.** Enrichment of eGenes in every quintile of the global dispersion ranking. Genes in the lowest quintile are depleted of eGenes (OR = 0.57, 95% CI: 0.33 - 0.92, *P* < 0.05). **B.** Comparison between low-dispersion and high-dispersion genes of hub gene rankings from the shared transcriptional networks of heart and skeletal muscle tissue (*P* < 10^−15^). **C.** Comparisons between low-dispersion and high-dispersion genes of per-gene connections in cell type-specific transcriptional networks. Left panel: cardiac fibroblasts (*P* < 0.01); Right panel: cardiomyocytes (*P* < 0.05). **D.** Comparison between low-dispersion and high-dispersion genes of per-gene interactions at the protein level (*P* < 10^−15^).

To test this, we used eQTLs mapped using cardiac-HDC single-cell RNA sequencing data from a sample of 46 individuals (Methods). These data allowed us to map eQTLs in the same cell types in which we estimated regulatory dispersion. We ranked genes by dispersion within each cell type and calculated, along the ranking (using a sliding-window approach; Methods), the proportion of genes associated with eQTLs (eGenes) identified in that cell type. Across cell types, low-dispersion genes were depleted for eGenes, whereas high-dispersion genes were enriched. This pattern was robust across analytical approaches: it was observed when dispersion rankings were analyzed separately within each cell type (Supplementary Figure S8), when the dispersion data from all cell types were combined (*P* < 0.05; Methods; Figure 3A), and even when eGenes were defined using multivariate adaptive shrinkage (MASH)^38^ to increase power for detecting eQTLs with small effect sizes (Methods; Supplementary Figure S9).

We next examined measures of functional pleiotropy. Pleiotropy has been shown to correlate with regulatory constraint. Connectivity within transcriptional and protein interaction networks serves as a proxy for functional pleiotropy. If low dispersion reflects higher regulatory fidelity and constraint, low-dispersion genes would be expected to exhibit greater connectivity.

To do this, we ranked all genes by their median dispersion across cell types (Methods). Genes at the top of this ranking represent those with consistently low dispersion across the studied cell types. We then considered three sources of data related to gene connectivity: (***i***) transcriptional co-expression networks for heart and skeletal muscle tissues derived from GTEx data^39^, (***ii***) cell type–specific co-expression networks for cardiomyocytes and cardiac fibroblasts derived from the same data set, and (***iii***) protein–protein interaction networks derived from data from the Human Protein Atlas (Methods).

Across all three datasets, we found that low dispersion genes are significantly more connected than high dispersion genes (Figures 3B-D; Supplementary Figure S10; *P* < 10^−15^ for comparisons of low and high dispersion genes in transcriptional co-expression networks for heart and skeletal muscle tissues, *P* < 0.05 for comparison in transcriptional co-expression cardiomyocytes and cardiac fibroblasts, and *P* < 10^−15^ for comparisons in protein-protein networks).

### Regulatory architecture differs between low- and high-dispersion genes

Given the associations between dispersion and measures consistent with regulatory fidelity, we next examined regulatory features that may underlie differences in gene expression dispersion. We first examined regulatory plasticity. Here, we define plasticity as variation in gene expression across tissues, which represent distinct regulatory environments. Under the hypothesis that lower regulatory fidelity is associated with increased plasticity^40,41^, genes with higher dispersion would be expected to show greater variation in expression across tissues.

**Figure 4.**
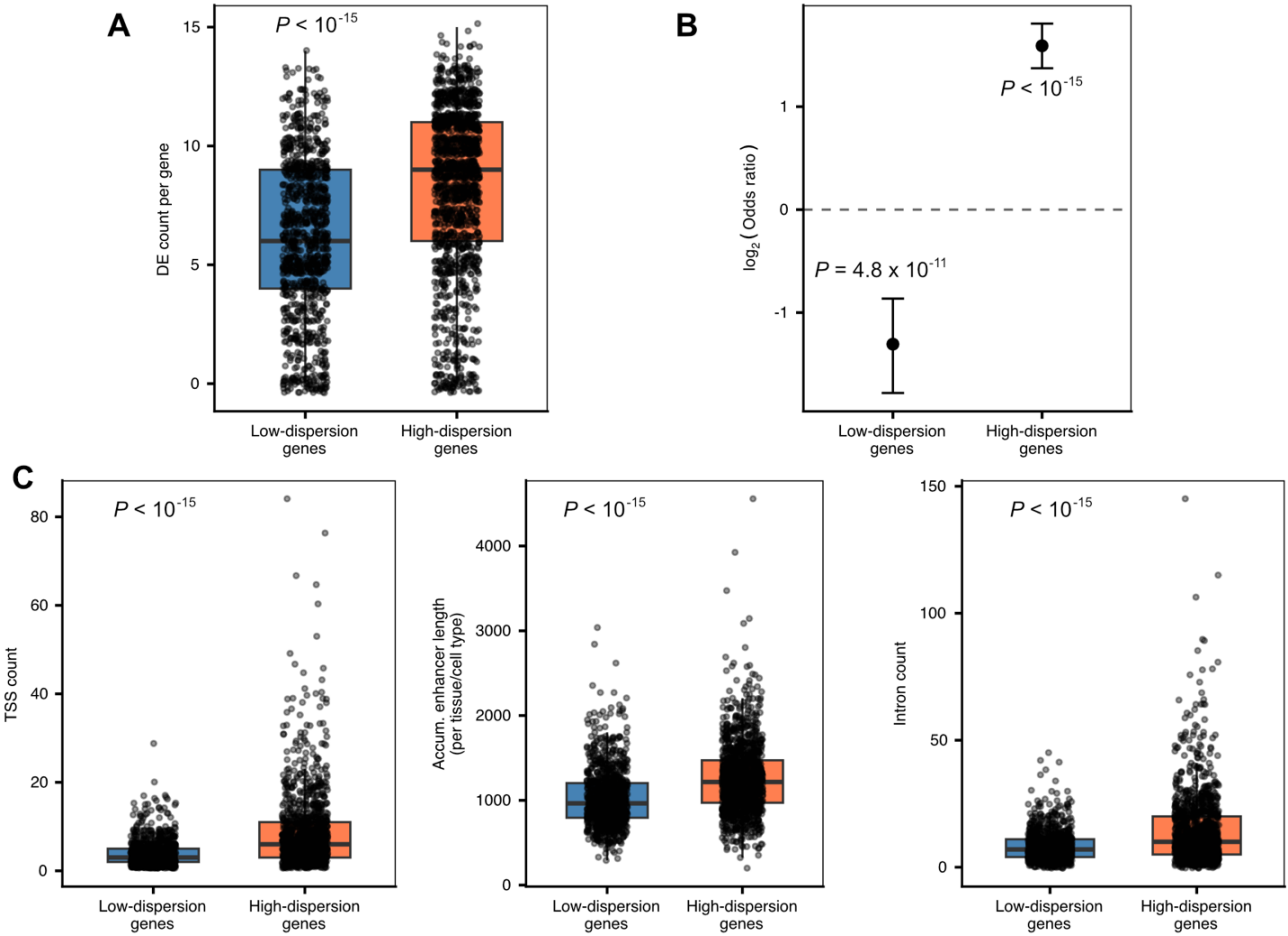
Regulatory architectures differ between low-dispersion and high-dispersion genes. **A.** Comparison of the number of pairwise tests in which low-dispersion and high-dispersion genes are differentially expressed (*P* < 10^−15^). **B.** Enrichment tests of TATA-containing promoters in low-dispersion and high-dispersion genes (low-dispersion genes: OR = 0.47, 95% CI: 0.34 – 0.62, *P* = 4.8 × 10^−11^; high-dispersion genes: OR = 2.93, 95% CI: 2.53 - 3.40; *P* < 10^−15^). **C.** Comparison of the number of transcription start sites (left panel), cumulative enhancer lengths (middle panel), and number of introns (right panel), between low-dispersion and high-dispersion genes.

We used bulk estimates based on the single cell data to identify differentially expressed genes between the cell types (Methods; Supplementary Table S3). Across the entire dataset, high-dispersion genes (top quintile) are classified as differentially expressed between cell types (at FDR < 0.05) more often than low-dispersion genes (bottom quintile; *P* < 10^−15^; Figure 4A). This observation is robust with respect to the statistical cutoff used to classify genes as differentially expressed, and the specific cell type comparisons that are being considered.

We next considered different aspects of regulatory architecture. We found that high-dispersion genes are more likely to be associated with TATA-containing promoters (OR = 2.93, 95% CI: 2.53 - 3.40) than low-dispersion genes (OR = 0.47, 95% CI: 0.34 – 0.62; *P* < 0.001; Figure 4B). This observation is consistent with previous reports^22,42^. We further found that high-dispersion genes are associated with a higher number of transcription start sites per gene (*P* < 10^−15^; Figure 4C; see Methods), and a longer cumulative associated enhancer length (*P* < 10^−15^; Figure 4C; see Methods). Finally, we compared intron counts per gene based on gene annotations (Methods) and found that high-dispersion genes have significantly more introns than low-dispersion genes (*P* < 10^−15^; Figure 4C).

### Gene expression dispersion is often shaped by *cis*-regulatory architecture

Having established that dispersion is structured and under genetic control, we next asked whether gene expression dispersion is regulated by *cis*- or *trans*-acting elements. We addressed this question using gene expression data that were collected in a comparative system in which interspecies regulatory differences can be partitioned into *cis* and *trans* components.

We analyzed previously generated single-cell RNA-seq data from unguided HDCs from three humans, three chimpanzees, and one human–chimpanzee allotetraploid line^43^. In the allotetraploid line, the human and chimpanzee genomes are present in the same nucleus. The human and the chimpanzee alleles, therefore, share the same *trans* environment. Using this study design, differences identified between the diploid human and chimpanzee lines that persist between the human and chimpanzee alleles in the allotetraploid line, indicate that the underlying regulatory divergence is encoded in *cis*, reflecting allele-specific effects. In contrast, differences observed between species in the diploid lines that are not recapitulated in the allotetraploid line indicate that the regulatory divergence is mediated in *trans*, arising from factors that operate through the shared cellular environment.

We estimated mean-corrected dispersion for each gene within each species and cell type, as described above. For each cell type, we identified genes with significant differences in dispersion between human and chimpanzee diploid lines (differentially dispersed or DD genes; Methods). Specifically, for each gene within a cell type, we fit a linear mixed model with species as a fixed effect and individual as a random effect (Methods). In the allotetraploid setting, we used Memento to perform differential dispersion analysis between species, as the experimental setup is optimized for a two-sample comparison (Methods). To increase power to detect DD genes, we then used the resulting DD effect sizes and standard errors as inputs for MASH^38^, which leverages shared patterns of effects across conditions to improve statistical power.

**Figure 5.**
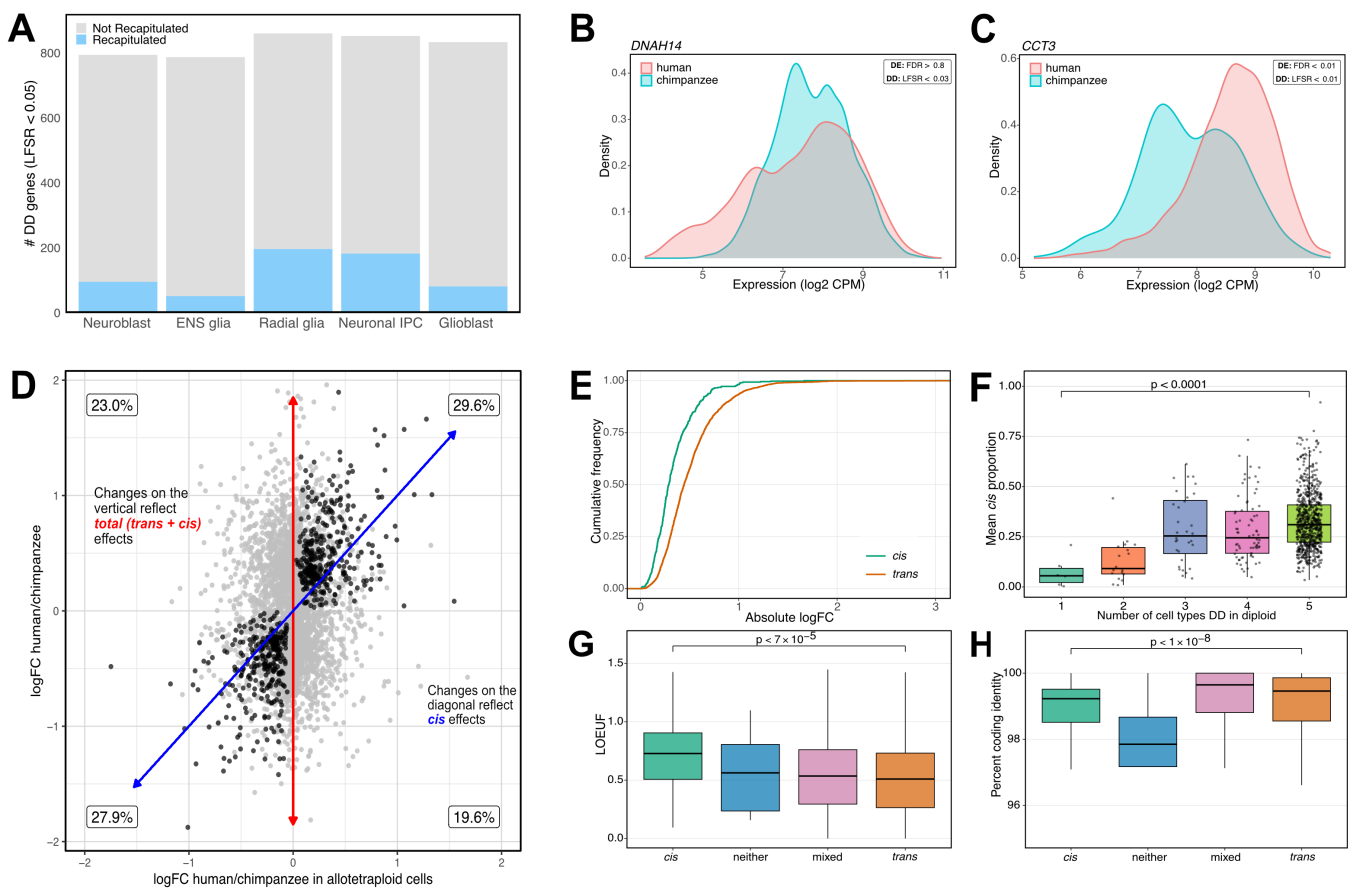
Interspecies differences in regulatory dispersion. **A.** The number of differentially dispersed (DD) genes (LFSR < 0.05) between humans and chimpanzees in diploid lines is shown in grey. The subset significantly (LFSR < 0.05) recapitulated in allotetraploid cells (regulated in *cis*) is shown in blue. **B.** Example of a DD gene that is not differentially expressed (DE) between the species but is differentially dispersed. **C.** Example of a DD gene with higher expression but lower dispersion between species. **D.** DD effect sizes for all cell types between humans and chimpanzees in diploid cells (y-axis) versus allotetraploid cells (x-axis). In each quadrant we note the percentage of DD genes. Genes with concordant effect size sign in diploid and allotetraploid cells are shown in black (though this definition underestimates potentially concordant *trans* effects). **E.** Empirical cumulative distributions of effect size for all cell types in diploid cells for tests with high *cis* proportion (> 0.70) and low *cis* proportion (< 0.30). **F.** Boxplot distribution of *cis* proportion for genes DD in the diploid cells for an increasing number of cell types. **G-H.** Boxplots showing the distribution of LOEUF and human-chimpanzee divergence (percent coding identity) respectively, for genes whose interspecies DD can be accounted for by a high, moderate, or low *cis* effect.

Across cell types, we identified between 787 and 860 interspecies DD genes per cell type (at local false sign rate (LFSR) < 0.05; median 833 DD genes per cell type; Figure 5A; Supplementary Tables S3–S4). Across cell types, 7% to 23% of interspecies DD genes identified in the diploid comparison were recapitulated as DD between the human and chimpanzee alleles in the allotetraploid line (Figure 5A; Supplementary Table S4). Thus, for an appreciable subset of genes with interspecies differences in dispersion, the difference persists in a shared *trans* environment. These observations indicate that interspecies differences in gene expression dispersion are often encoded by *cis*-regulatory sequence divergence between the two species.

We highlight two patterns of DD genes of particular interest that deviate from the expected mean-variance relationship in scRNA-seq data^44,45^. For instance, in radial glia cells, *DNAH14*, which encodes heavy chain axonemal dyneins^46^, exhibits comparable expression between species despite significant differences in dispersion (Figure 5B). In glioblast cells, *CCT3*, a gene involved in the folding of various proteins such as actin and tubulin^47^, shows higher mean expression but lower dispersion in humans compared to chimpanzees (Figure 5C). Together, these patterns indicate that gene expression dispersion reflects a regulatory feature that is distinct from changes in mean expression.

To quantify *cis* effects more continuously, we compared the interspecies dispersion effect size between the diploid and allotetraploid lines (Methods). We used this comparison to estimate, for each gene in each cell type, the proportion of diploid DD effect size that is recapitulated in the allotetraploid line between the human and chimpanzee alleles (Figure 5D). When the proportion of diploid DD effect size was below 30% or exceeded 70%, we classified the difference in dispersion between the species as arising from interspecies differences in predominantly *trans* or *cis* elements, respectively (Methods; Supplementary Figure S12; Supplementary Table S5).

We characterized the differences between *cis*- and *trans*-driving DD genes. We found that when interspecies differences in dispersion are driven predominantly by *trans*-acting elements, the DD effect size between species is larger than when interspecies differences in dispersion are driven predominantly by *cis*-acting elements (*P* < 10^−11^; Figure 5E). However, interspecies differences in dispersion driven predominantly by *trans*-acting elements tend to be more cell-type specific than interspecies DD driven by *cis* (*P* < 0.0001; Figure 5F). Genes associated with interspecies differences in dispersion driven predominantly by *trans*-acting elements are less tolerant to loss of function mutations than genes where interspecies DD is driven by *cis* (*P* < 7 × 10^−5^; Figure 5G) and have lower coding region divergence between human and chimpanzee (percent ID; *P* < 10^−8^; Figure 5H). These patterns were robust with respect to a range of *trans* and *cis* definitions and cutoffs (Supplementary Figures S13–S16).

## Discussion

Gene expression variability has long attracted attention from multiple perspectives. Mechanistic studies established that transcription often occurs in bursts^48^, with promoter switching, polymerase recruitment, and RNA processing kinetics generating cell-to-cell differences in mRNA abundance^6,16,49,50^. In these studies, expression variability was often seen as a byproduct of different transcriptional mechanisms. In parallel, population-level studies showed that gene expression variability across individuals can change following perturbations, such as immune challenge, even when mean expression does not^27^. In these studies, variability was generally measured across individuals, not between individual cells.

Here, we frame expression variability, and more specifically, dispersion, as a phenotype that reflects regulatory fidelity. We hypothesized that regulatory dispersion is structured, genetically encoded, and functionally patterned. The evidence supports this view. Dispersion in gene expression shows reproducible, gene-specific structure across cell types. Genes involved in core cellular processes exhibit consistently low dispersion, whereas genes associated with cell-type–specific programs display context-dependent dispersion. Dispersion correlates with regulatory constraint, network connectivity, and promoter and enhancer architecture. Interspecies comparisons further show that a measurable fraction of interspecies difference in expression dispersion is encoded in *cis*-regulatory sequence. Together, these observations indicate that dispersion, or fidelity, is a dimension of gene regulation subject to genetic control.

The structural and functional features associated with dispersion clarify its biological meaning. High-dispersion genes are more frequently differentially expressed across cell types, consistent with greater regulatory plasticity. These genes are enriched for TATA-containing promoters, multiple transcription start sites, longer enhancer landscapes, and increased intron number. Low-dispersion genes are depleted for these features and enriched for housekeeping functions and network connectivity. Dispersion therefore lies along a fidelity–plasticity axis. This notion is consistent with previous observations^40,41^. Genes requiring stable maintenance of expression exhibit high fidelity and narrow distributions. Genes that must remain responsive to distinct regulatory environments exhibit greater structural complexity and broader distributions. Promoter and enhancer architecture appear to shape not only bursting kinetics but also the tolerated breadth of expression variability.

The distinction between mechanistic origin and regulatory function is central. Transcriptional bursting may provide the molecular substrate for variability^51^. The degree to which variability is constrained; however, differs systematically across genes and contexts and is subject to natural selection. Dispersion thus reflects regulatory pressure related to precision. Some genes require tight control across individual cells.

Several caveats merit consideration. Dispersion is estimated in our study within clusters inferred to represent homogeneous cell types or states. Imperfect clustering is generally expected to inflate dispersion as unrecognized heterogeneity would increase rather than decrease the observed variability. Conclusions regarding low dispersion are therefore conservative. To partly mitigate this concern, we evaluated dispersion across multiple clustering resolutions and a reference-based assignment strategy^36^. The principal patterns are robust to these approaches.

In the comparative system, *trans*-associated differences in dispersion may capture both genetic and non-genetic components of the cellular environment. While the comparative system enables unambiguous identification of *cis* contributions, *trans* effects likely reflect a mixture of regulatory divergence and broader context. Nonetheless, the *trans*-acting elements we identified as contributing to interspecies differences in dispersion tend to have larger effect sizes and greater cell-type specificity. This pattern is consistent with previous findings for mean gene expression levels^52,53^ and suggests that a substantial fraction of these *trans* effects are likely to be real. *Trans* regulatory mutations are expected to be more pleiotropic and therefore more likely to be deleterious. Consequently, the *trans* regulatory mutations that persist are not a random sample, but a selected subset enriched for variants that were advantageous, or in some cases effectively neutral. This survivorship bias predicts that fixed *trans* effects may be relatively large, but confined to fewer contexts, where pleiotropic costs are minimized.

Recognizing dispersion as regulatory fidelity expands how transcriptional phenotypes are conceptualized. Mean expression defines the target level of transcription. Dispersion defines the precision with which that level is achieved across cells. Changes in dispersion can alter the fraction of cells that cross functional thresholds without altering the mean. Such effects are invisible in pseudobulk analyses.

This perspective has direct implications for threshold-dependent phenotypes, including disease onset, drug response, developmental commitment, and gene–environment interactions. Outcomes traditionally attributed to shifts in mean expression may instead reflect changes in regulatory fidelity. A narrow distribution concentrates cells near the mean. A broader distribution increases the probability that some cells cross a functional boundary. Whether increased dispersion enhances or diminishes a phenotype depends on the position of the mean relative to the threshold. If the mean lies below a boundary, greater dispersion increases the chance of crossing it. If the mean lies above it, greater dispersion increases the chance of falling below it. Dispersion is therefore neither inherently beneficial nor detrimental; its effect depends on context.

This framework also helps reconcile prior observations in developmental systems. Studies of transcriptional bursting during differentiation have reported that increased variability can be associated either with enhanced plasticity^54,55^ or with reduced stability of terminal states^56^. When variability is viewed solely as a mechanistic byproduct, these interpretations appear inconsistent. When variability is interpreted as regulated fidelity that modulates threshold crossing, both outcomes become plausible. Dispersion can facilitate transitions when a population approaches a threshold from one side and destabilizes states when a population must remain beyond it.

In summary, gene expression dispersion is structured, genetically controlled, shaped by *cis*- and *trans*-regulatory architecture, and aligned with a fidelity–plasticity continuum. Variability is not simply noise generated by transcriptional bursting. It is a regulated property of gene expression that influences how cellular populations approach and cross functional boundaries.

## Methods

### Experimental model and study design

#### Cardiac HDC Model

We differentiated and sampled 3 induced pluripotent stem cell lines from unrelated Yoruba individuals from Ibadan, Nigeria (YRI). We maintained cell lines on Matrigel Growth Factor Reduced (GFR) Basement Membrane (354230, Corning) with Essential 8 medium (A1517001, Thermo Fisher Scientific) and Penicillin-Streptomycin (17–602F, Lonza). Cells were grown at 37°C and 5% CO2 to roughly 80% confluency. Thereafter, we passaged cell cultures with a dissociated reagent (0.5 mM EDTA, 300 mM NaCl in PBS) and seeded cells with ROCK inhibitor Y-27632 (ab120129, Abcam).

We differentiated cardiac HDCs from iPSCs following the procedure developed in the Gilad lab^35^. On the 12^th^ day of culture in the differentiation medium, we collected and dissociated cardiac HDC aggregates as previously described. Following dissociation, we counted cells and recorded viability using Trypan Blue and Countess II (AMQAF1000, Invitrogen). We then mixed cells from each cell line into a single suspension in equal proportions and centrifuged cells at 100 g for 3 minutes. Finally, we resuspended cells in 4°C base heart medium for single-cell RNA seq library preparation.

#### Human-Chimpanzee HDC Model

We used existing single-cell RNA sequencing data from Barr et al.^52^, which consists of 3 humans, 3 chimpanzees, and 1 human-chimpanzee allotetraploid line. An unguided differentiation approach^57^ was used to form each line into HDCs (i.e., without enriching for cardiac cell types). We applied CrossFilt^58^ to the raw published dataset to eliminate species alignment bias. Next, we used the clustering-free multivariate statistical method CelliD^59^ to extract gene expression signatures of individual cells. We then annotated cells based on enrichment of these signatures, along with marker genes and cell atlas references^60,61^. We included cell types that had at least 1,000 cells in human and chimpanzee diploid lines in our analysis to ensure sufficient power to detect differential dispersion (Supplementary Figure S11).

### Single-cell RNA-sequencing

With the cells collected from our cardiac HDCs, we performed single-cell RNA sequencing (scRNA-seq) with the 10X Genomics Chromium GEM-X Single Cell 3’ Reagents Kits v4 (Dual Index) (PN-1000215, 10X Genomics). We prepared libraries in a single batch. With 7 lanes of a 10X chip, we recovered roughly 30,000 cells per lane. We sequenced the libraries with the Illumina NovaSeq X at the University of Chicago Functional Genomics Core facility, yielding approximately 87,329 mean reads per cell. We aligned reads to the human reference genome (GRCh38) using CellRanger (v8.0.1) and demultiplexed individuals with Vireo (v0.5.6)^62^. We performed downstream processing analyses in R (v4.4.1) / RStudio (v2024.4.2.764) with Seurat (v5.2.1)^63^. We removed cells classified as doublets or unassigned according to Vireo, and we filtered for cells with at least 4,500 expressed genes. These filtering steps resulted in a total of 38,606 expressed genes and 152,223 cells across the three cell lines.

After filtering, we proceeded to cell type annotation. As noted, the cardiac HDC model was previously developed in the Gilad lab, and cell type annotations were rigorously defined both manually with marker gene expression and against multiple reference datasets^35^. Thus, to annotate our cells, we reference mapped to the annotated data in the previous report^35^. We first log normalized raw counts and selected the 5,000 most variable genes with Seurat’s Variance Stabilizing Transformation method (vst). We then scaled variable features with Seurat’s ScaleData function with default settings. After scaling, we applied principal component analysis (PCA), taking the top 50 principal components, and performed clustering with the Louvain algorithm as implemented in Seurat’s FindClusters function at a resolution of 0.2. With clustered cells and a shared reduction space (PCA) between our dataset and the reference data, we performed the reference mapping procedure in Seurat. Briefly, with the top 50 principal components in both the reference and query datasets, we used FindTransferAnchors to define transfer anchors as cells that are mutual nearestneighbors between the datasets. Then, we used the TransferData function to classify our dataset according to the cell type annotations in the reference^64^. Finally, we applied uniform manifold approximation and projection (UMAP) to the data for ease of visualization.

### Calculation of mean and dispersion (residual variance)

We computed gene expression mean and residual variance (residual variance represents the mean-corrected variance or dispersion/variability) with Memento^65^ for each cell type that contained at least 1,000 cells in each individual for the cardiac HDC model. We computed these moments within clusters of cells represented by both cell type and individual. Thus, we made three measurements for each gene in each cell type (3 individuals). To select for genes that are sufficiently expressed for reliable measurement of residual variance, Memento internally filters out genes with a mean raw UMI count less than 0.07 on a per cluster basis. We set the capture rate (i.e., overall detection efficiency) parameter to *q* = 0.4 to reflect the efficiency of our measurements. Further, to avoid spurious distinctions between cell types driven by power differences, we chose to keep only genes that were expressed above this threshold in all cell type + individual clusters. These filtering steps left us with over 10,000 measured genes.

We applied the same framework in the human-chimpanzee HDC model to estimate mean expression and dispersion. Specifically, estimates were computed for each species, cell type, individual, and replicate in diploid lines, and for each species, cell type, and replicate in the allotetraploid line. For these analyses, we used a more conservative capture rate of *q* = 0.2.

### Gene ontology enrichment analysis

We performed gene ontology enrichment (GO) analysis using the R package clusterProfiler^66^. clusterProfiler computes enrichment tests for a set of genes among GO terms or other biological ontologies based on the hypergeometric distribution. Here, we tested gene sets from pairwise DD tests and rank-based analyses (see below) against GO Biological Process (GO:BP) gene sets within the Molecular Signatures Database^67^ (MSigDB v2025.1.Hs).

We defined background gene sets differently for DD test and rank-based gene sets. For gene sets from DD tests, we were interested in testing low DD genes with respect to all high DE genes. Thus, we defined background genes as high DE genes (FDR < 0.05 and positive log fold change) that overlap with genes in GO:BP gene sets. For rank-based GO enrichments, we defined background genes as those that overlapped between genes in GO:BP gene sets and all measured genes in the ranked gene sets. We corrected for multiple testing with the Benjamini-Hochberg procedure and an FDR cutoff ≤ 0.05 and q-value cutoff ≤ 0.2.

In a supplementary analysis, we performed the rank-based GO enrichment test described above for two alternative clustering resolutions (Supplementary Figure S5). Thus, rank-based GO enrichments were performed at resolutions 0.2, 0.05, and 0.08 as defined in the FindClusters Seurat function (Supplementary Table S2)^63^.

### Gene ranking and rank-based analyses

#### Gene Ranking

For all analyses that used rankings of gene expression dispersion, we ranked genes within cell types. There are three measurements per gene per cell type (three individuals for each cell type) in the cardiac HDC data, so we used the median dispersion value per gene across individuals to produce cell type-specific gene rankings.

#### Defining low-dispersion and high-dispersion genes

We considered low- and high-dispersion genes in two contexts for this study: (1) low- and high-dispersion across cell types, and (2) low- and high-dispersion within cell types. Most analyses involved the first context. Here, we binned each cell type-specific dispersion ranking into quintiles. We defined low-dispersion genes as genes in the first quintile (lowest dispersion) across every cell type considered. We defined high-dispersion genes as genes in the fifth quintile (highest dispersion) across every cell type considered.

For low- and high-dispersion genes defined within cell types, we used the lowest ranked 30 percent and highest ranked 30 percent of genes, respectively. In supplementary analyses, we also tested cutoffs at 10 percent and 20 percent (Supplementary Figure S10).

#### Distribution of low-dispersion genes along mean expression ranking

To confirm that the shared dispersion properties of the consistently low-dispersion and high-dispersion genes were not driven by shared mean expression, we plotted the distributions of these genes with respect to mean expression. We ranked genes by the median of their mean expression values across cell types, then binned them into quintiles. Within each quintile, we counted the number of low-dispersion and high-dispersion genes, respectively (Supplementary Figure S4).

### Differential dispersion and differential expression analyses

#### Cardiac HDC Model

We performed differential variability analysis between cell types with data from the single batch of cardiac HDCs. We first quantile normalized the residual variance measurements described in the last section. For each sample (cell type and individual) we set cell type as a fixed effect in a standard linear model. Using dreamlet^68^, we fit the model to each gene and defined a contrast matrix representing all pairwise cell type comparisons for hypothesis testing. Finally, we applied empirical Bayes shrinkage and used the Benjamini-Hochberg procedure^69^ to identify differentially dispersed (DD) genes as those with an FDR-adjusted *P* value < 0.05.

We applied the same procedure to test differential mean expression (DE), identifying DE genes with an FDR-adjusted *P* value < 0.05.

#### Variance partition analysis

We performed variance partition analysis with the variancePartition R package^70^. We tested for the contribution of both biological and technical factors. Following the standard procedure described in the variancePartition documentation, we fit a linear mixed model where cell type was considered as random effects, and cell number, number of features, and percent of mitochondrial reads per sample were considered as fixed effects.

#### Human-Chimpanzee HDC Model

To perform differential variability analysis between species in diploid cell lines, we first estimated variability as dispersion using Memento for each sample (cell type, individual, and replicate). Next, we quantile normalized dispersion and fit a linear mixed-effects model separately for each gene and cell type using dreamlet, with species as a fixed effect and individual as a random effect. Effect sizes and standard errors from dreamlet were input into MASH^71^, a Bayesian method that models sharing across conditions to improve power and effect size estimates. Significant interspecies DD genes in the diploid line were identified using a local false sign rate (LFSR) < 0.05. We performed differential variability analysis between species in the allotetraploid line using Memento, which is well calibrated to perform hypothesis testing in a two-sample comparison. In an allotetraploid cell, both human and chimpanzee DNA exist in the same *trans* environment. This creates an ideal setting for direct comparisons of variability differences without confounding from batch effects. Similarly, effect sizes and standard errors from Memento were input into MASH to improve power to detect DD genes. We identified significant DD genes as genes with an LFSR < 0.05 in both diploid and allotetraploid settings and the same sign of effect size.

To perform DE analysis between humans and chimpanzees in diploid cells, we generated pseudobulk expression profiles for each sample (defined by cell type, individual, and replicate) by summing counts across cells. We then used dreamlet to fit a linear mixed-effects model for each gene within each cell type, with species included as a fixed effect and individual as a random effect. For allotetraploid cells, we performed a paired Wilcoxon signed-rank test comparing, within each cell, the number of reads assigned to human versus chimpanzee alleles. We implemented this test using the row_wilcoxon_paired function from the matrixTests R package^72^. For both analyses, *P* values were adjusted using the Benjamini–Hochberg procedure.

### eGene enrichment analysis

#### cardiac HDC collection and eQTL mapping

eQTL data was previously collected in McIntire et al. (unpublished results from cardiac HDCs). Here, we will briefly describe the data collection and eQTL mapping processes.

We differentiated and collected cells from cardiac HDCs from 49 YRI lines using the same cell culture, cardiac HDC differentiation, and collection protocols described above. We sequenced cells from all 49 lines using the 10X Genomics Chromium Next GEM Single Cell 3’ HT Reagent Kits v3.1 (Dual index, PN-1000348, 10X Genomics). We performed alignment, demultiplexing, filtering, and clustering as described above, with some key differences. First, we removed genes expressed in fewer than three cells. Second, we filtered out cells with fewer than 1,500 genes. Following quality control, three individuals were removed, resulting in a final dataset of 46 individuals. We performed cell type annotation as described in McIntire et al.^35^ Finally, for downstream eQTL mapping, we performed pseudobulking within samples (cells represented by cell type and individual). We only kept individuals for pseudobulking in each cell type if they contained at least 15 cells within that cell type. Further, we only kept cell types that contained at least 25 individuals.

We applied linear modeling in MatrixEQTL to map *cis*-eQTLs for each sample. We mapped *cis*-eQTLs with whole-sequencing data from the 1000 Genomes Project, testing SNPs for associations with gene expression within 100kb windows of transcription start sites (TSSs). We excluded SNPs with a minor allele frequency below 0.05.

In a supplementary analysis (Supplementary Fig S9) described below, we relied on eGenes identified with MASH. For this set of eGenes, the underlying linear model applied in MatrixEQTL tested SNPs within a 500kb window and included covariates for sex, batch, and cell line passage number at the time of differentiation.

#### Cell type-specific eGene sliding window enrichment analysis

We performed the following analysis first with eGenes identified using MatrixEQTL^73^, then again with eGenes identified after MASH implementation to increase power. We first ranked all measured genes by dispersion within cell types, then, we calculated proportions of eGenes within sliding windows across the dispersion rankings. To compute 95% confidence intervals, for each window we randomly sampled a set of genes equal to the number of eGenes in a given cell type, then calculated the proportion of that gene set represented in the window of ranked genes. We conducted this sampling procedure 50 times for each window.

We restricted the analysis with MatrixEQTL to eGenes with an eQTL discovered at a Benjamini-Hochberg-corrected FDR < 0.05. Further, we tested only four cell types: cardiomyocytes, activated fibroblasts, cardiac fibroblasts, and foregut endodermal cells (an insufficient number of eGenes were detected for this analysis in epithelial and mesenchymal cells). After implementing MASH, a LFSR< 0.05 was used to identify eGenes and all cell types were tested.

#### Global eGene enrichment

The global eGene enrichment analysis differed from the cell type-specific analysis only in the selection of measured genes to rank and eGenes to test. Since we aimed to test for a cell type-agnostic relationship between regulatory variation and dispersion, we limited our analysis to genes with a relatively stable dispersion ranking across cell types, which we identified by computing the median ranking of each gene across four cell types and setting a 15 % threshold that defined how far a gene’s rank could deviate from the median within a cell type. If a gene did not deviate beyond this threshold in any cell type, it was kept for the global enrichment analysis. We then binned these globally ranked genes into quintiles and tested them for enrichment in the eGenes previously identified with MatrixEQTL at an FDR < 0.05, using Fisher’s exact test to quantify enrichment in each quintile. Given the insufficient number of eGenes detected in epithelial and mesenchymal cells, this analysis excluded them both in the ranking of genes and pooling of eGenes.

### Other features relating to the functional significance of dispersion / variability

We compared regulatory characteristics including transcription network complexity and protein interactions between high-dispersion and low-dispersion genes. For analyses on transcription network complexity, we used tissue-specific transcriptome-wide networks (TWNs) and related-tissue TWNs from Saha et al.^39^. We only considered interactions of total expression (TE) levels between genes. For tissue-specific TWNs, we compared the number of TE-TE connections between cell type-specific high-dispersion and low-dispersion genes. For related-tissue TWNs, we compared the ranking of hub genes between high-dispersion and low-dispersion genes across cell types; a lower hub gene ranking indicates more TE-TE connections. Finally, we performed a similar analysis comparing the number of protein interactions per gene between high- and low-dispersion genes, using protein interaction data from the Human Protein Atlas^74–77^.

### Estimates of *cis* proportion

We estimated the proportion of *cis* and *trans* contributions to gene expression variability divergence by comparing interspecies DD effect sizes of genes in the allotetraploid line to the corresponding effect size in the diploid lines. Taking the absolute value of the log2 fold change between humans and chimpanzees, we calculated the *cis* proportion as abs(allotetraploid.logFC)/(abs(allotetraploid.logFC) + abs(diploid.logFC − allotetraploid.logFC)). This is an adaptation of a procedure used by several previous studies to estimate *cis/trans* contribution to mean expression levels^52,78,79^. We classified a gene as *cis* within a cell type if the estimated *cis* proportion is greater than 70%; if less than 30%, we classified it as *trans*; otherwise, the genes were classified as *neither* (Supplementary Figure S12). Genes classified as *cis* in one cell type and *trans* in another were labeled as *mixed*. Otherwise, genes were classified as *cis* or *trans* if they were assigned that category in at least one cell type and were categorized as *neither* or not DD in all remaining cell types. Genes were classified as *neither* if they were categorized as *neither* in at least one cell type and were not DD in all others. Analyses using stricter definitions, which yielded fewer clear *cis* and *trans* cases, are shown in the supplement (Supplementary Table S5; Supplementary Figures 13–16).

### Characterization of regulatory architecture and constraint

We compared genic features including intron counts, transcription start site (TSS) counts, and per gene cumulative enhancer lengths between genes that were high-dispersion and genes that were low-dispersion across cell types. For intron counts, we used the Ensembl BioMart^80^ and counted introns according to the Matched Annotation from NCBI and EMBL-EBI (MANE) select transcript^81^ for each gene. We calculated TSS counts per gene according to the promoter peaks identified in Cap Analysis of Gene Expression (CAGE) experiments and compiled in the FANTOM5 database^82^. Finally, we determined per-gene enhancer lengths by calculating the mean cumulative enhancer length across 131 tissues and cell types as predicted by an Activity-by-contact (ABC) model^83^ (ABC score > 0.05). We obtained the percent coding identity between humans and chimpanzees from BioMart^84^, using chimpanzee as the reference. To characterize the intolerance of interspecies DD genes to loss of function mutations, we obtained loss-of-function observed/expected upper bound fraction (LOEUF) scores from a study of 141,456 human exomes and used scores according to the MANE select transcript with option = ‘true’ for each gene^85^.

### Correlations and *P* values

Correlations reported in the main text were computed using Spearman’s rank-order correlation tests implemented using the R function *corr.test* with option method = ‘s’. To compare effect sizes of *cis* and *trans* effects, a *P* value was calculated using a Kolmogorov-Smirnov test with the R function *ks.test*. For all analyses comparing differences between distributions, we computed *p*-values using the two-sided Mann–Whitney *U* test with the R function *wilcox.test*. To test for enrichments in the eGene and TATA enrichment analyses, we used Fisher’s exact test.

## Supplementary Material

Supplement 1

Supplement 2

Supplement 3

4

## Data Availability

The single-cell RNA-seq data have been deposited at GEO under accession GSE326116. All original code, data, and summary statistics presented in this paper have been deposited on GitHub (https://github.com/awchen55/dispersion).
