# Supplementary material for: Beyond the mean: genetic control of gene expression fidelity and dispersion
Source: bioRxiv. 2026 Apr 7:2026.04.01.715853. Preprint. [Version 2] doi: 10.64898/2026.04.01.715853 (PMC13082100; doi:10.64898/2026.04.01.715853)
Supplement: 4 [file NIHPP2026.04.01.715853v2-supplement-4.pdf]

bioRxiv preprint doi: https://doi.org/10.64898/2026.04.01.715853; this version posted April 7, 2026. The copyright holder for this preprint (which was not certified by peer review) is the author/funder, who has granted bioRxiv a license to display the preprint in perpetuity. It is made available under aCC-BY 4.0 International license.

Supplementary Figures

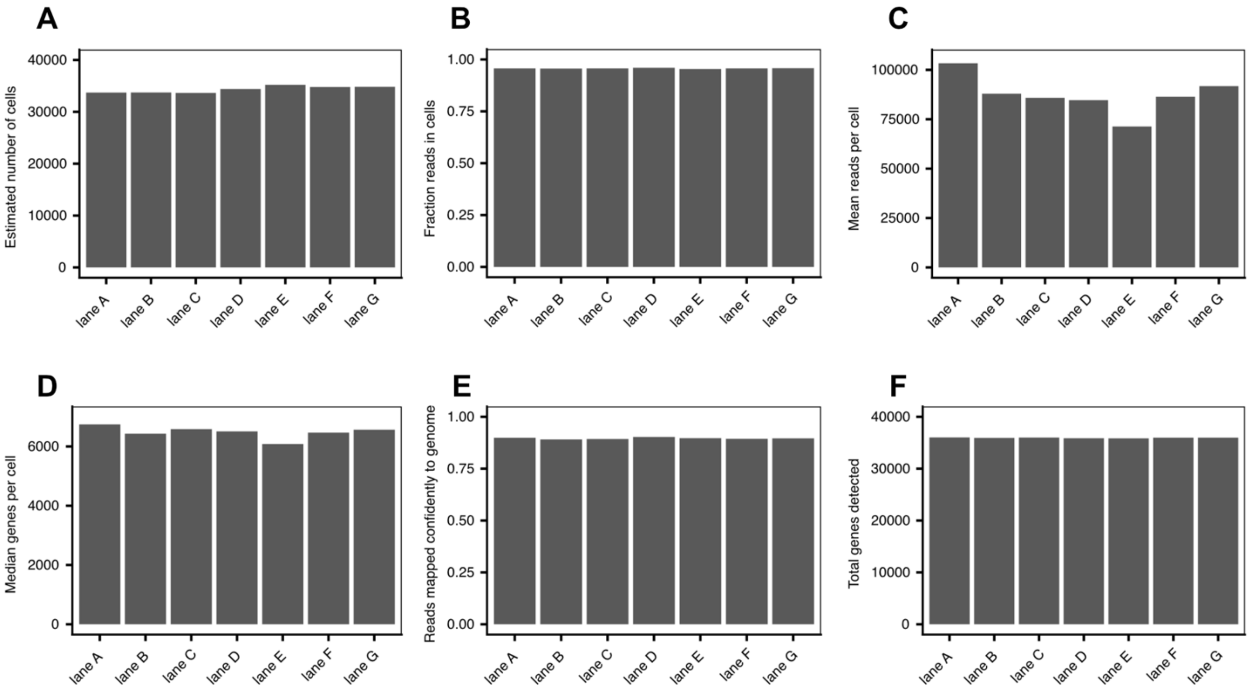

Figure S1. Quality control metrics for each single cell RNA-seq library. **A** Estimated number of cells per lane. **B** Proportion of reads in cells per lane. **C** Mean reads per cell per lane. **D** Median number of detected genes per cell per lane. **E** Proportion of genes mapped confidently to the genome per lane. **F** Total number of genes detected per lane.

bioRxiv preprint doi: https://doi.org/10.64898/2026.04.01.715853; this version posted April 7, 2026. The copyright holder for this preprint (which was not certified by peer review) is the author/funder, who has granted bioRxiv a license to display the preprint in perpetuity. It is made available under aCC-BY 4.0 International license.

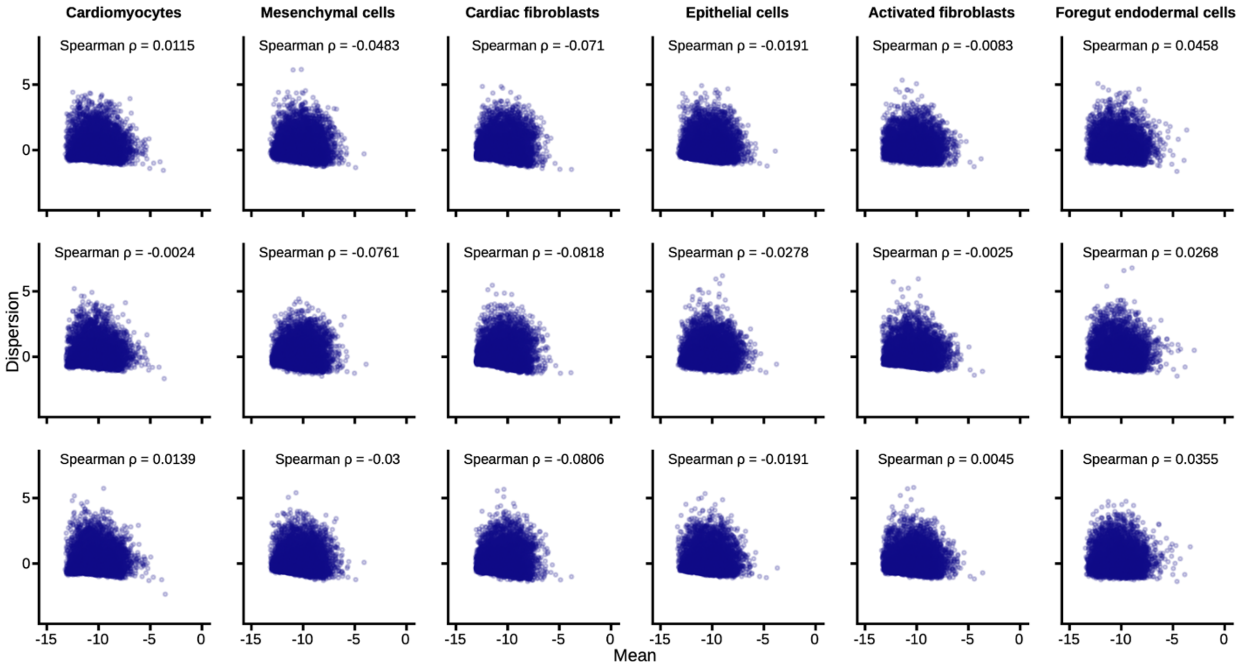

Figure S2. Mean-independence of dispersion. Mean effect on dispersion tested for each sample. Dispersion and mean expression estimates were natural log transformed. Spearman correlation coefficient reported for each sample.

bioRxiv preprint doi: https://doi.org/10.64898/2026.04.01.715853; this version posted April 7, 2026. The copyright holder for this preprint (which was not certified by peer review) is the author/funder, who has granted bioRxiv a license to display the preprint in perpetuity. It is made available under aCC-BY 4.0 International license.

A

For cell *i* and gene *j:*

$$y_{ij}|\lambda_{ij} \sim Pois(L_i\lambda_{ij}) \quad (1)$$

$$\lambda_{ij} \sim f(\cdot) = \begin{cases} Gamma(\mu_j, \phi_{j_0}) & \text{for background gene} \\ Gamma(\mu_j, \phi_j) & \text{for dispersed gene} \end{cases} \quad (2)$$

Equation 1: The Poisson distribution is the measurement model, where y_{ij} represents the number of unique molecular identifiers (UMI) and is dependent on the parameters: L_i (library size) and λ_{ij} (latent expression value). **Equation 2:** The Gamma distribution is the expression model, where λ_{ij} depends on the mean, μ, and the dispersion parameter, ϕ. Under this framework, the background or null gene has a base level of dispersion represented as ϕ_{j_0}.

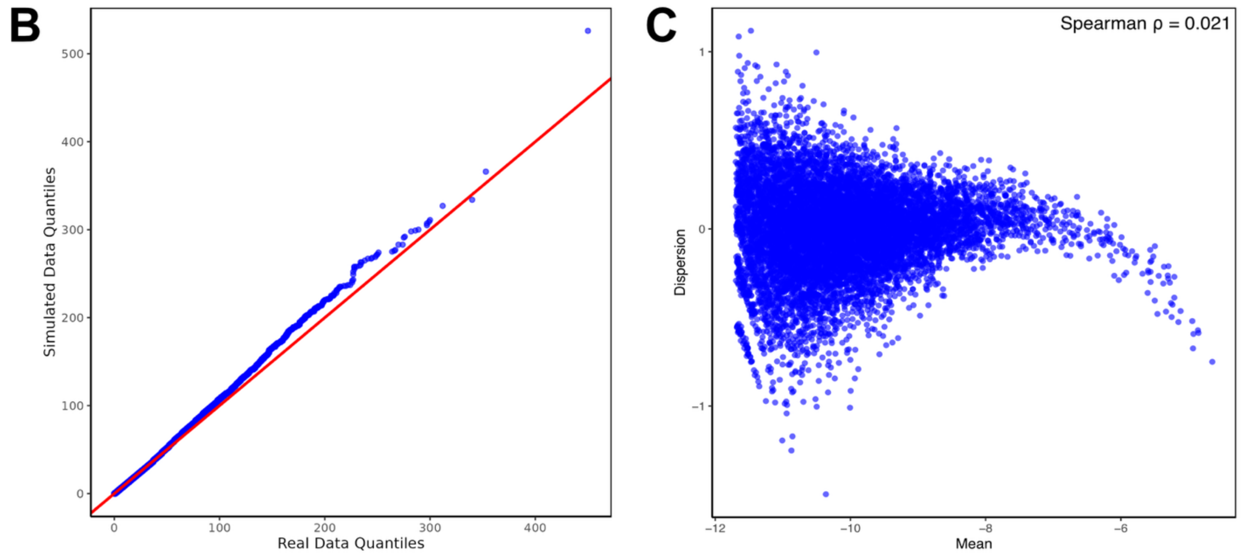

Figure S3. Mean-independence of dispersion using simulated data. **A** Gamma-Poisson framework used to simulate single-cell RNA sequencing data. Specifically, we used a single replicate of the human iPSC derived cardiomyocytes consisting of 546 single cells. Genes were first pooled into bins of mean expression. A Gamma-Poisson distribution was then fitted on each pooled bin using the R package, *glmGamPoi* (Ahlmann-Eltze and Huber, 2020), to estimate ϕ. The average ϕ across all bins was used to estimate ϕ_{j_0} and set as the background gene dispersion level. For each gene in the real data, we estimated μ_j. The average library size for cells in the real data was used for L_i. Given estimates for each real data genes' parameters, we simulated the expression and measurement models respectively using the base R functions *rgamma* and *rpois*. **B** Q-Q plot for simulated versus real data. **C** Mean effect on dispersion tested on the simulated data. Dispersion and mean expression estimates were natural log transformed. Spearman correlation coefficient is shown.

bioRxiv preprint doi: https://doi.org/10.64898/2026.04.01.715853; this version posted April 7, 2026. The copyright holder for this preprint (which was not certified by peer review) is the author/funder, who has granted bioRxiv a license to display the preprint in perpetuity. It is made available under aCC-BY 4.0 International license.

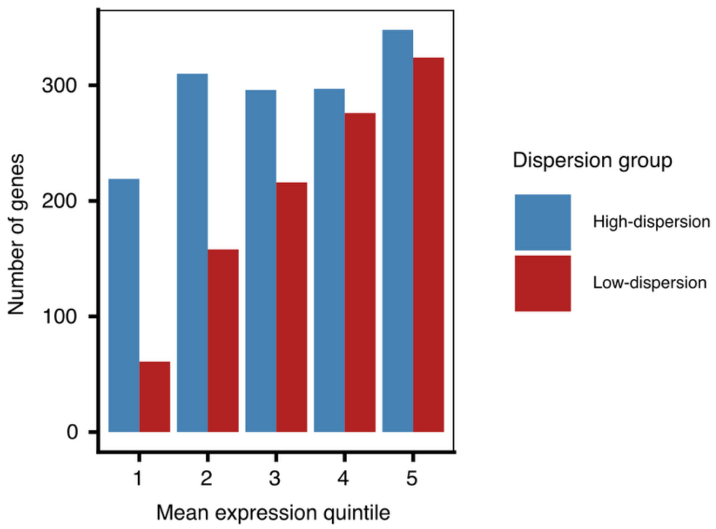

Figure S4. Distribution of high and low-dispersion genes across mean expression quintiles.

bioRxiv preprint doi: https://doi.org/10.64898/2026.04.01.715853; this version posted April 7, 2026. The copyright holder for this preprint (which was not certified by peer review) is the author/funder, who has granted bioRxiv a license to display the preprint in perpetuity. It is made available under aCC-BY 4.0 International license.

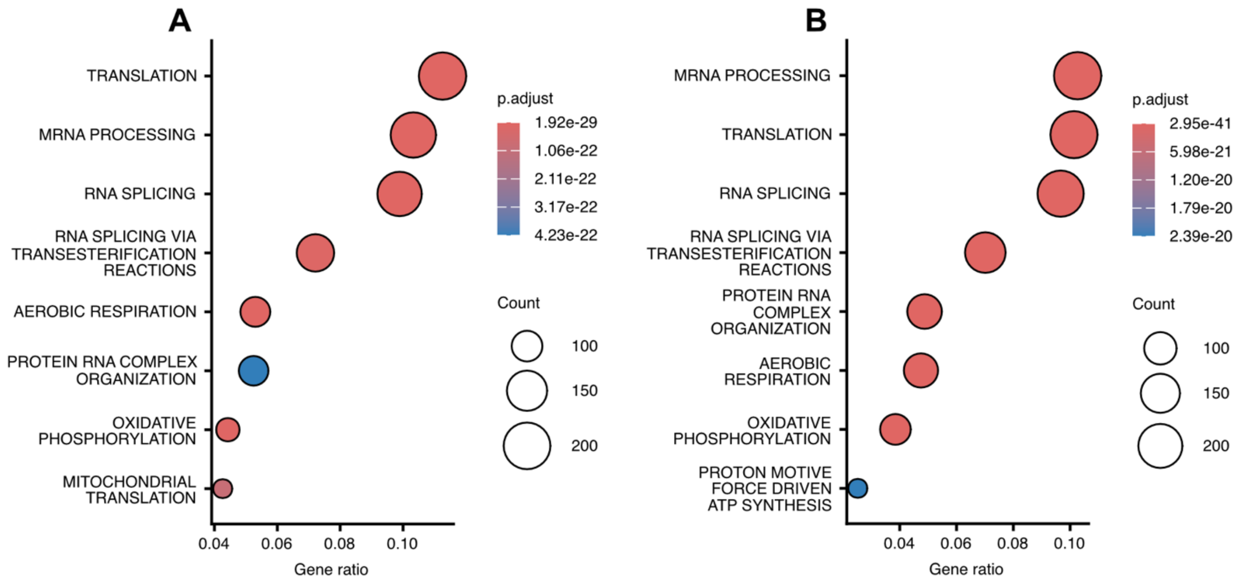

Figure S5. Gene ontology (GO) enrichments of low-dispersion genes in alternative clustering resolutions. **A** GO enrichment for genes shared in the first quintile of ranked dispersion across clusters at a resolution of 0.05 (Methods). **B** GO enrichment for genes shared in the first quintile of ranked dispersion across clusters at a resolution of 0.08 (Methods).

bioRxiv preprint doi: https://doi.org/10.64898/2026.04.01.715853; this version posted April 7, 2026. The copyright holder for this preprint (which was not certified by peer review) is the author/funder, who has granted bioRxiv a license to display the preprint in perpetuity. It is made available under aCC-BY 4.0 International license.

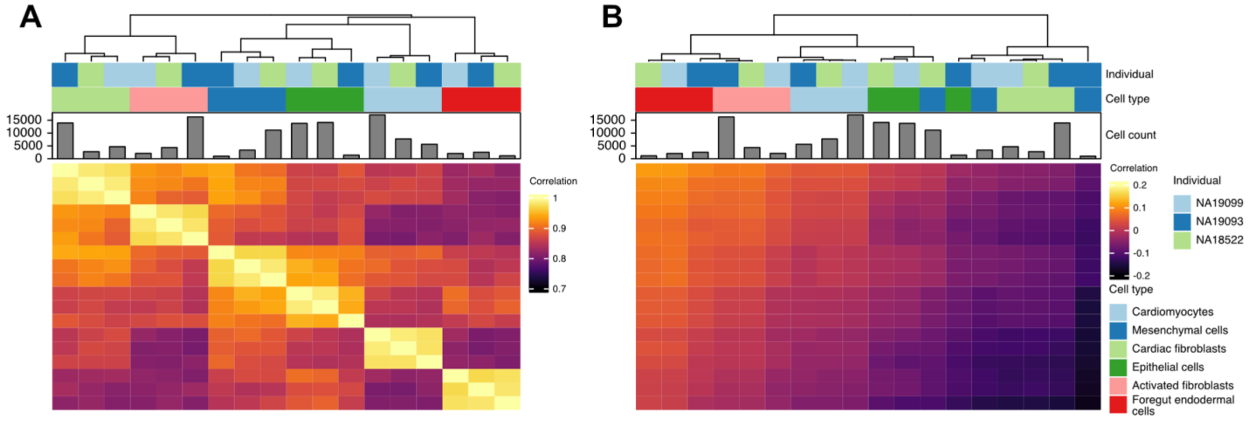

Figure S6. Clustering samples by mean expression and mean vs dispersion. **A** Correlation structure of mean expression profiles across samples. **B** Correlation structure of mean expression vs dispersion profiles for each sample.

bioRxiv preprint doi: https://doi.org/10.64898/2026.04.01.715853; this version posted April 7, 2026. The copyright holder for this preprint (which was not certified by peer review) is the author/funder, who has granted bioRxiv a license to display the preprint in perpetuity. It is made available under aCC-BY 4.0 International license.

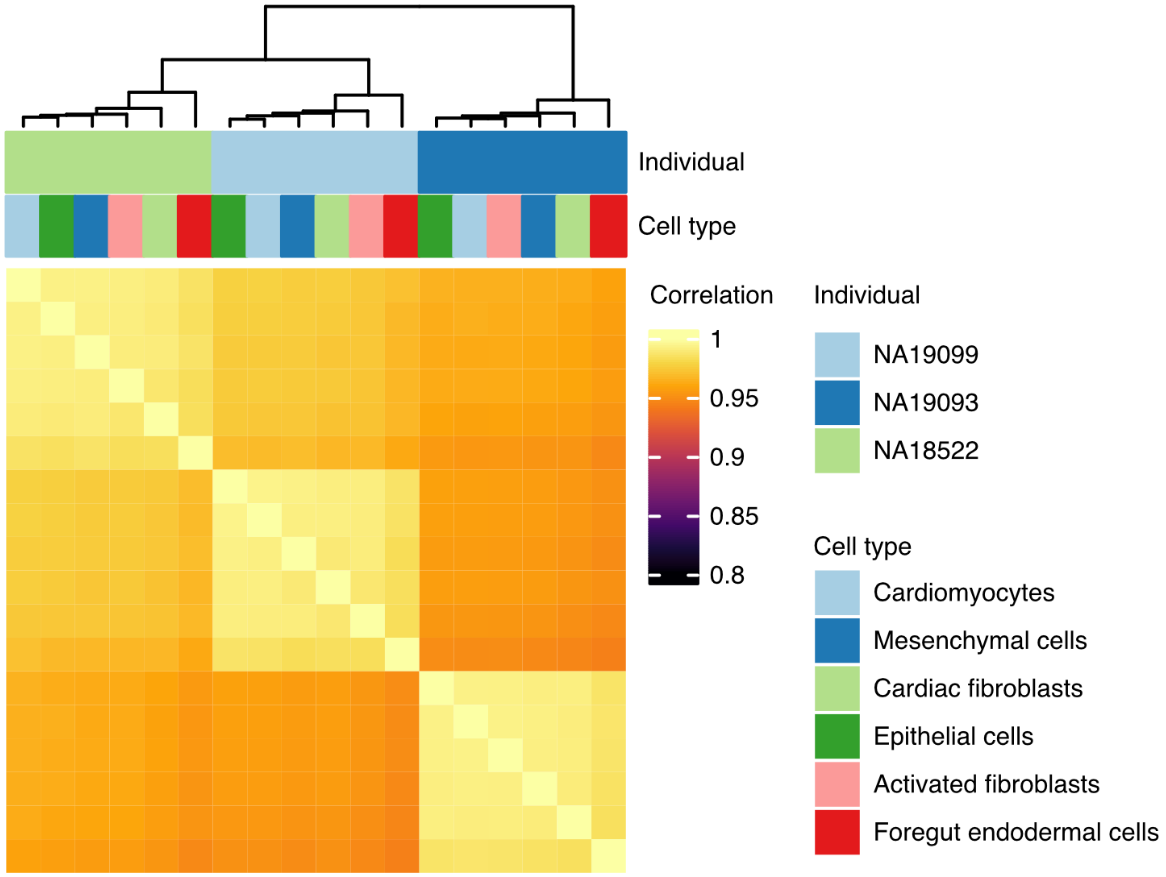

Figure S7. Correlation structure of dispersion profiles across samples following random permutation of cell type labels.

bioRxiv preprint doi: https://doi.org/10.64898/2026.04.01.715853; this version posted April 7, 2026. The copyright holder for this preprint (which was not certified by peer review) is the author/funder, who has granted bioRxiv a license to display the preprint in perpetuity. It is made available under aCC-BY 4.0 International license.

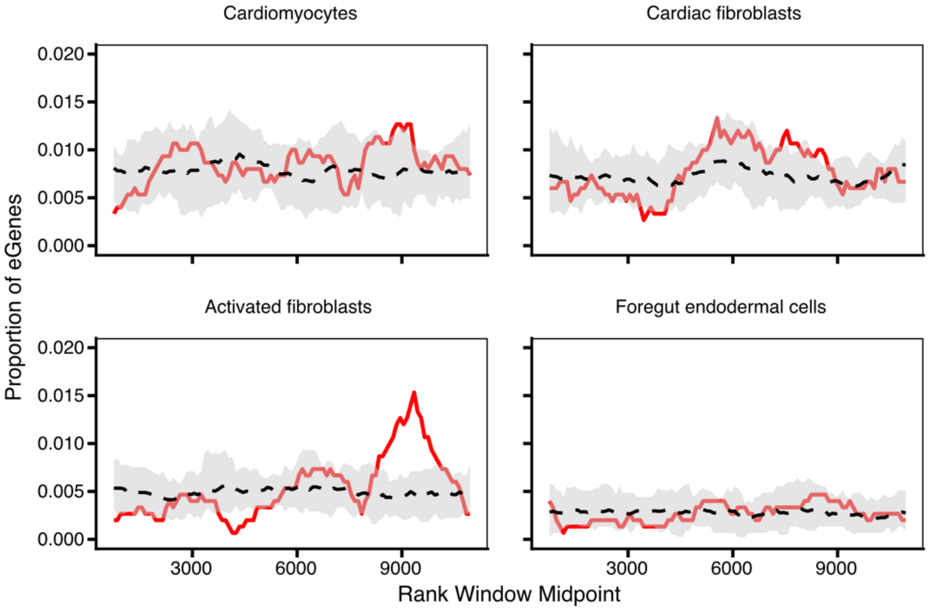

Figure S8. Enrichments of eGenes discovered with MatrixEQTL in sliding windows of genes ranked by dispersion in each cell types. The numbers per cell type are small – a couple dozen eGenes.

bioRxiv preprint doi: https://doi.org/10.64898/2026.04.01.715853; this version posted April 7, 2026. The copyright holder for this preprint (which was not certified by peer review) is the author/funder, who has granted bioRxiv a license to display the preprint in perpetuity. It is made available under aCC-BY 4.0 International license.

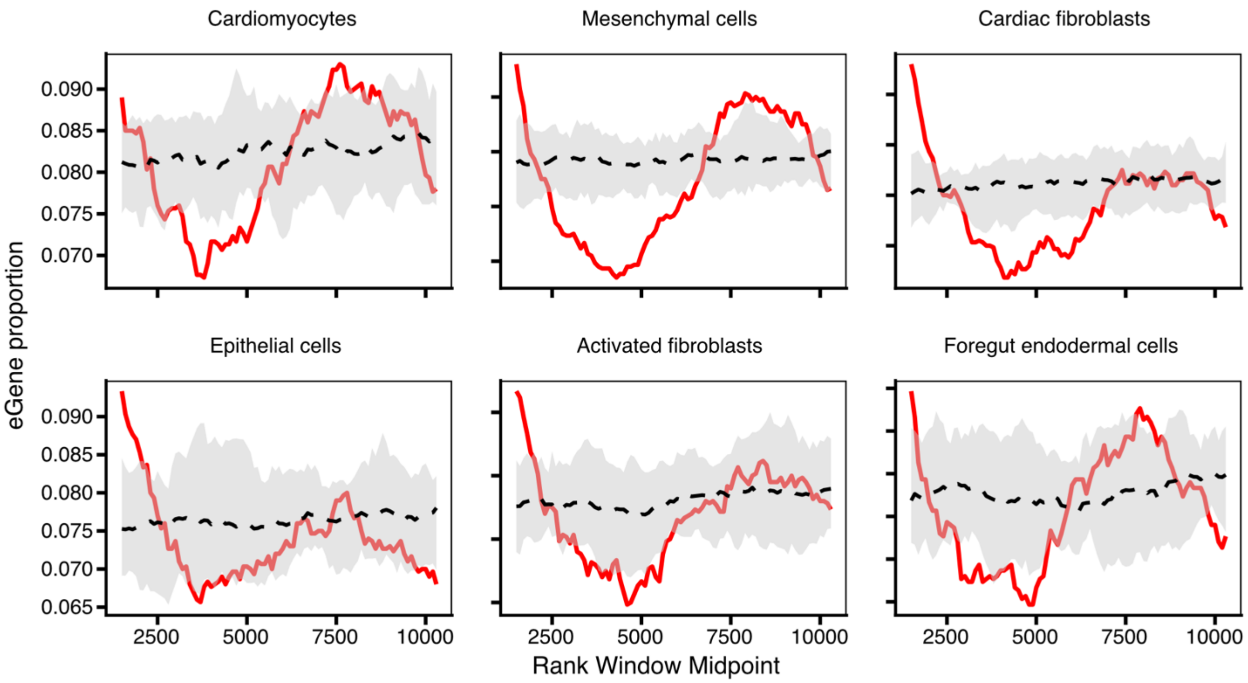

Figure S9. Enrichments of eGenes discovered with MASH in sliding windows of genes ranked by dispersion within cell types. MASH is conservative and results in considerable power to detect eQTLs. At the limits, where dispersion is very small or very large, the variance impacts power, which results in the enrichment and depletion of eGenes at the very tails.

bioRxiv preprint doi: https://doi.org/10.64898/2026.04.01.715853; this version posted April 7, 2026. The copyright holder for this preprint (which was not certified by peer review) is the author/funder, who has granted bioRxiv a license to display the preprint in perpetuity. It is made available under aCC-BY 4.0 International license.

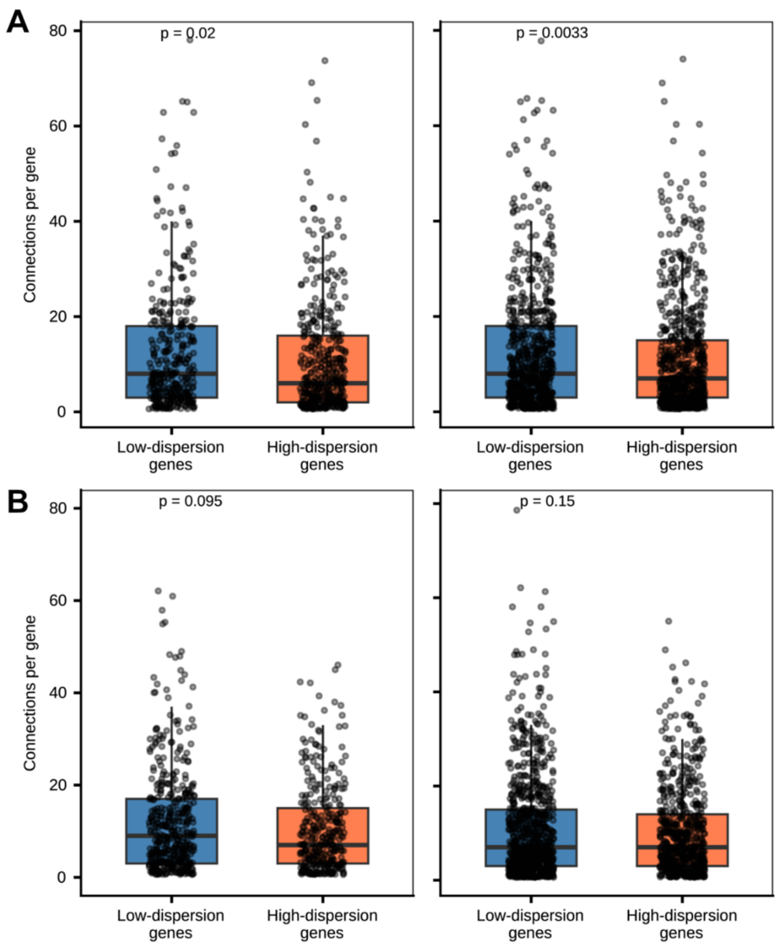

Figure S10. Robustness in comparison of per gene connections in cell type-specific co-expression networks. Multiple cutoffs for defining low and high-dispersion genes in cell type-specific dispersion rankings were used to test the robustness of the observation of greater connectivity in low-dispersion genes relative to high-dispersion genes. **A** Comparison of the number of connections per gene in the low-dispersion versus high-dispersion sets in cardiac fibroblasts. **B** Comparison of the number of connections per gene in the low-dispersion versus high-dispersion sets in cardiomyocytes. **Left panels:** Gene sets defined as the 10% tails of the cardiac fibroblast-specific ranking. **Right panels:** Genes sets defined as the 20% tails.

bioRxiv preprint doi: https://doi.org/10.64898/2026.04.01.715853; this version posted April 7, 2026. The copyright holder for this preprint (which was not certified by peer review) is the author/funder, who has granted bioRxiv a license to display the preprint in perpetuity. It is made available under aCC-BY 4.0 International license.

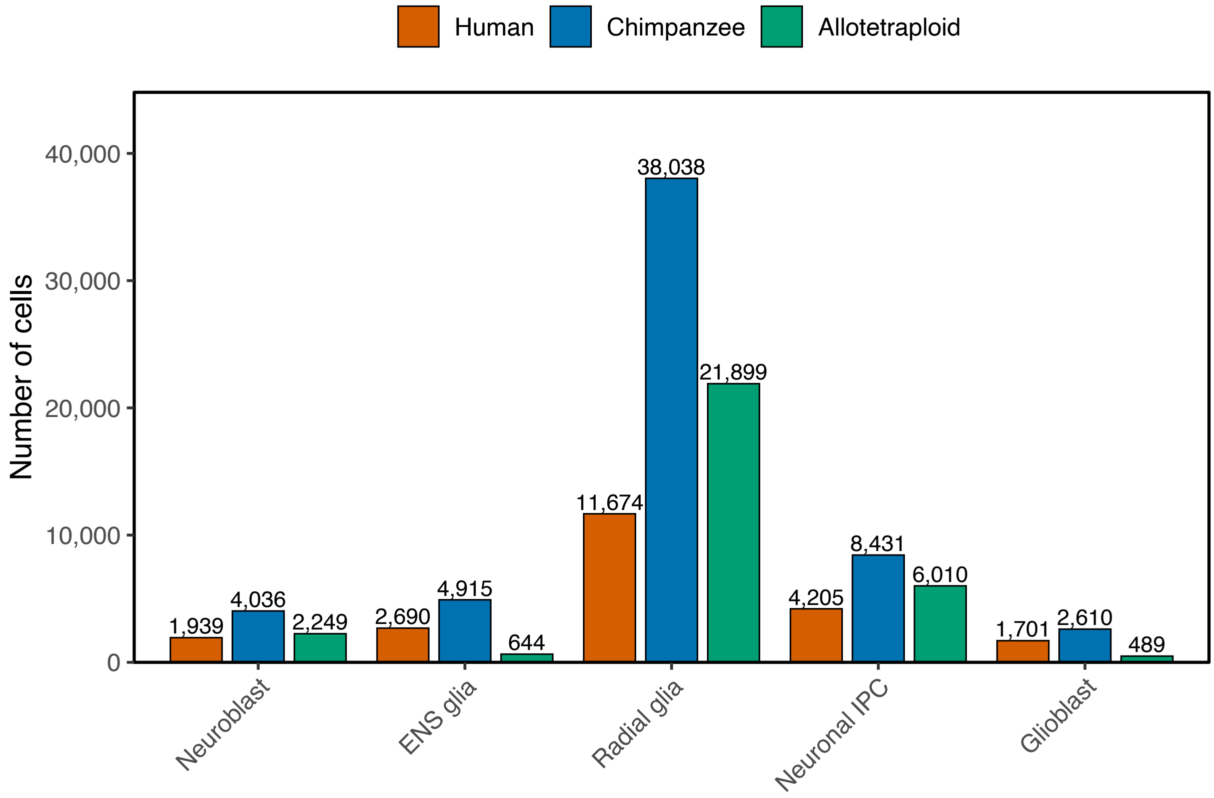

Figure S11. Number of single cells within each cell type for diploid human, diploid chimpanzee, and allotetraploid cells used in the interspecies analysis.

bioRxiv preprint doi: https://doi.org/10.64898/2026.04.01.715853; this version posted April 7, 2026. The copyright holder for this preprint (which was not certified by peer review) is the author/funder, who has granted bioRxiv a license to display the preprint in perpetuity. It is made available under aCC-BY 4.0 International license.

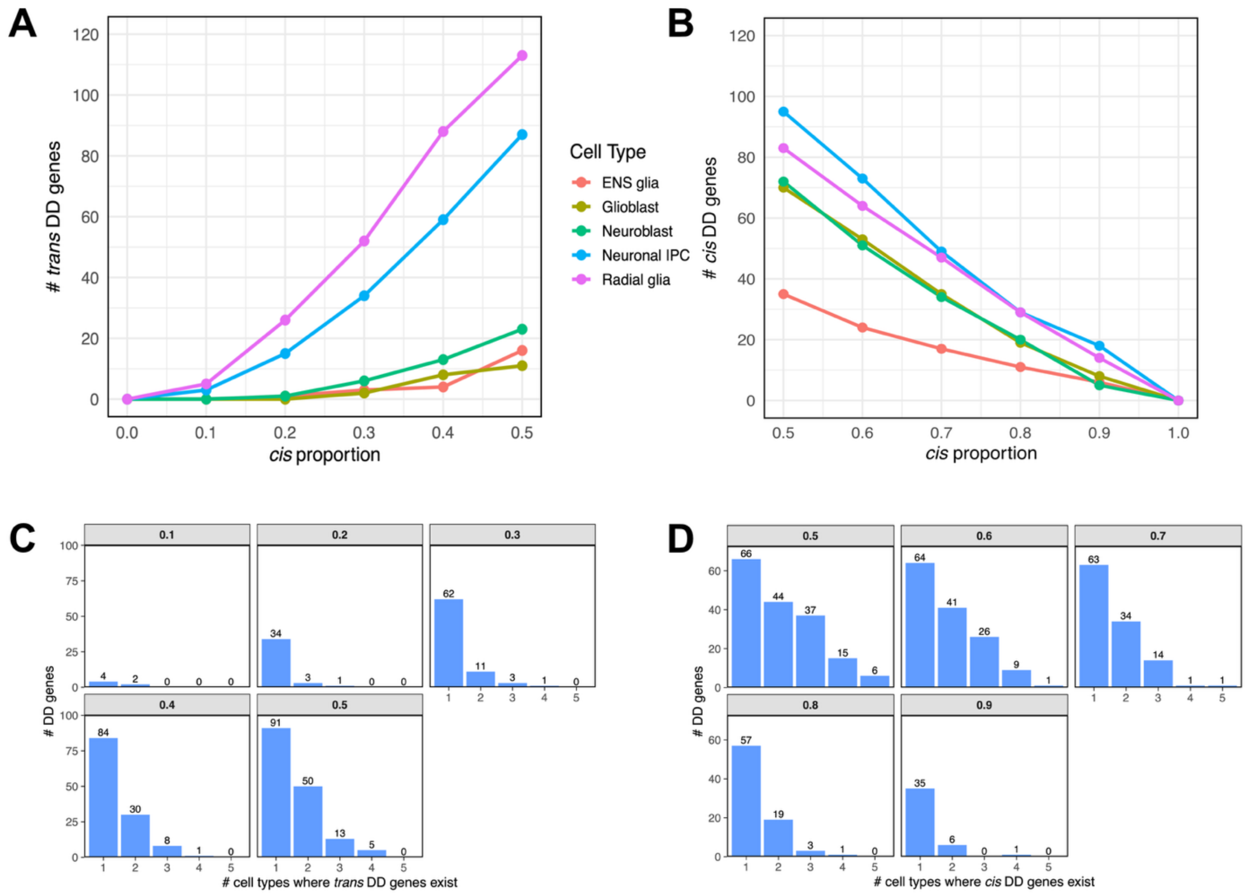

Figure S12. Number of *trans* and *cis* DD genes based on *cis* proportion cutoffs. **A-B** Among identified significant DD genes within a cell type (LFSR < 0.05; recapitulated in both diploid and allotetraploid cells), we classify *trans* or *cis* DD genes based on their estimated *cis* proportion. We vary the *cis* proportion threshold, defining *trans* DD genes as those with *cis* proportion below the cutoff and *cis* DD genes as those with *cis* proportion above the cutoff. **C-D** For each *cis* proportion cutoff, we quantify the number of DD genes that exist in multiple cell types, separately for *trans* and *cis* DD genes.

bioRxiv preprint doi: https://doi.org/10.64898/2026.04.01.715853; this version posted April 7, 2026. The copyright holder for this preprint (which was not certified by peer review) is the author/funder, who has granted bioRxiv a license to display the preprint in perpetuity. It is made available under aCC-BY 4.0 International license.

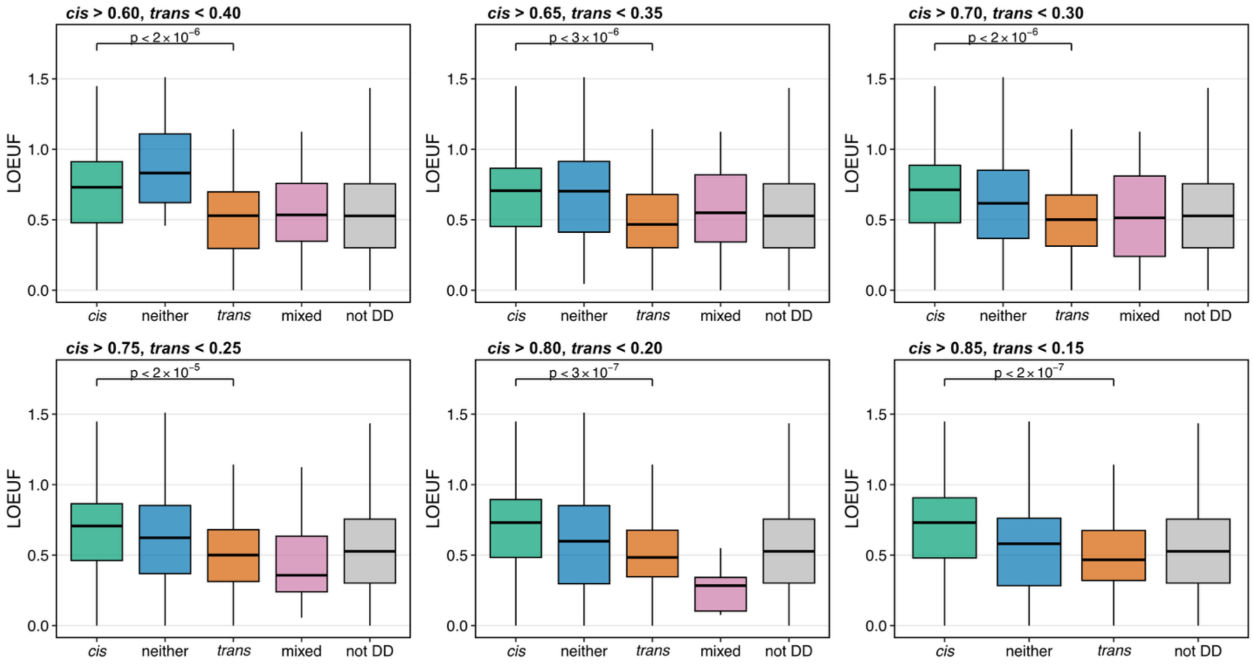

Figure S13. LOEUF scores based on different *cis* proportion cutoffs to define *cis* and *trans* DD genes, considering data from all cell types.

bioRxiv preprint doi: https://doi.org/10.64898/2026.04.01.715853; this version posted April 7, 2026. The copyright holder for this preprint (which was not certified by peer review) is the author/funder, who has granted bioRxiv a license to display the preprint in perpetuity. It is made available under aCC-BY 4.0 International license.

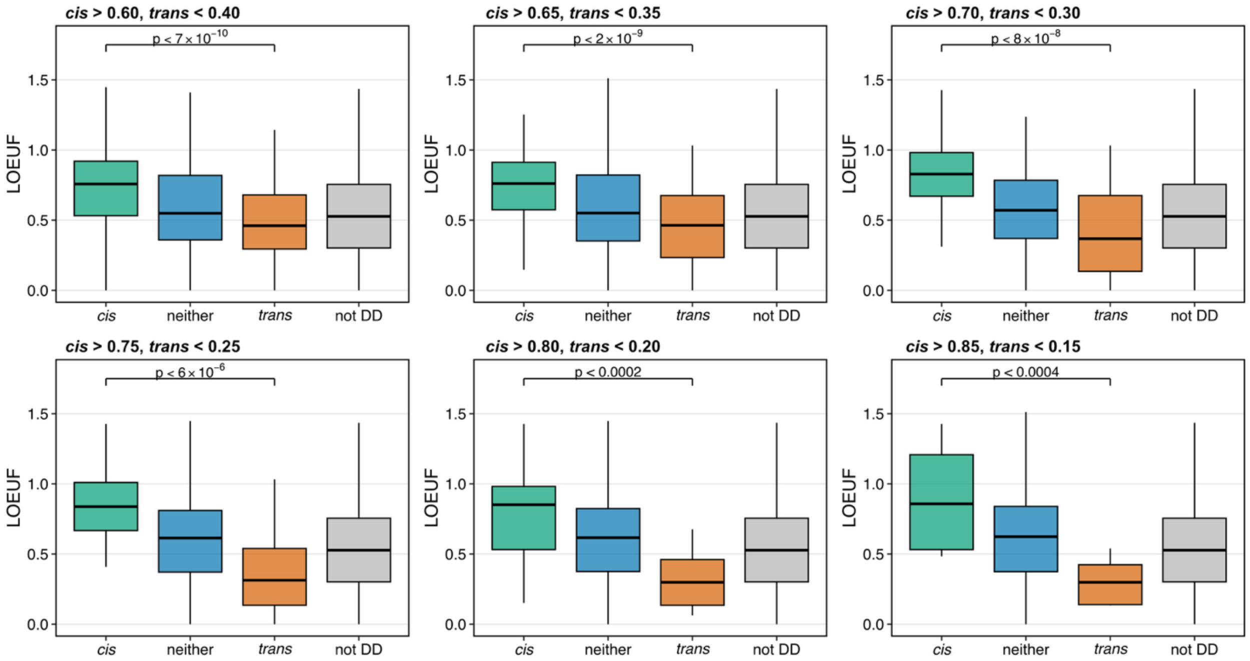

Figure S14. LOEUF scores based on different mean *cis* proportion cutoffs of cell types DD to define *cis* and *trans* DD genes, across cell types.

bioRxiv preprint doi: https://doi.org/10.64898/2026.04.01.715853; this version posted April 7, 2026. The copyright holder for this preprint (which was not certified by peer review) is the author/funder, who has granted bioRxiv a license to display the preprint in perpetuity. It is made available under aCC-BY 4.0 International license.

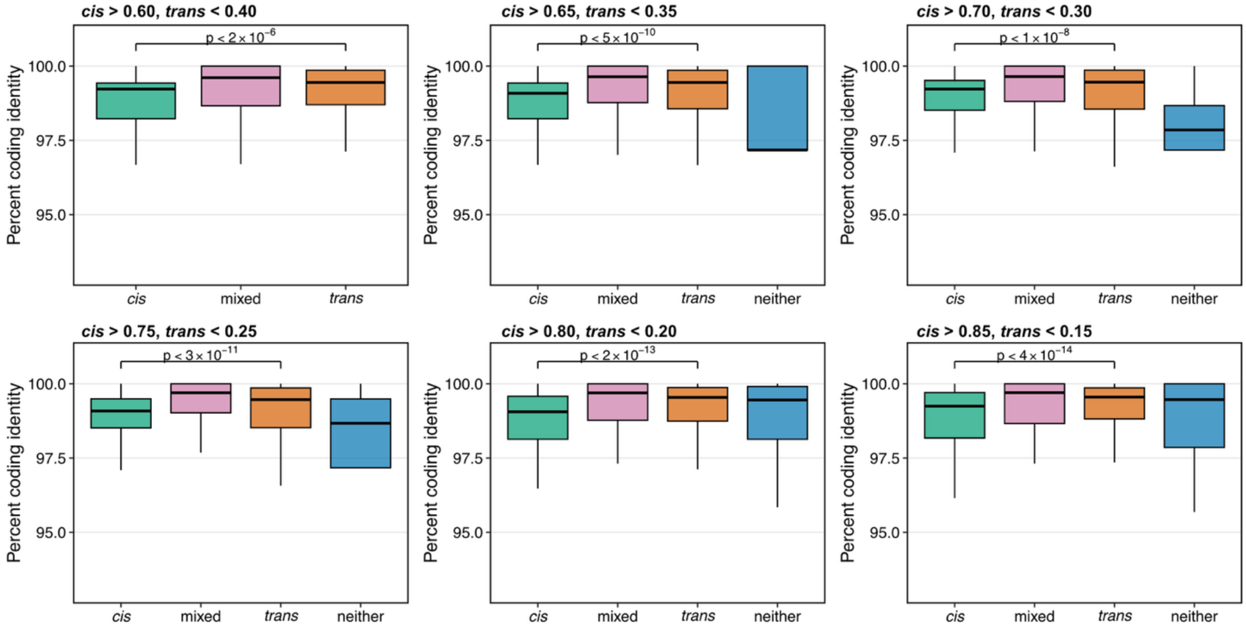

Figure S15. Percent coding identity (human-chimpanzee divergence) based on different proportion *cis* contribution cutoffs to define *cis* and *trans* DD genes when data from all cell types are considered.

bioRxiv preprint doi: https://doi.org/10.64898/2026.04.01.715853; this version posted April 7, 2026. The copyright holder for this preprint (which was not certified by peer review) is the author/funder, who has granted bioRxiv a license to display the preprint in perpetuity. It is made available under aCC-BY 4.0 International license.

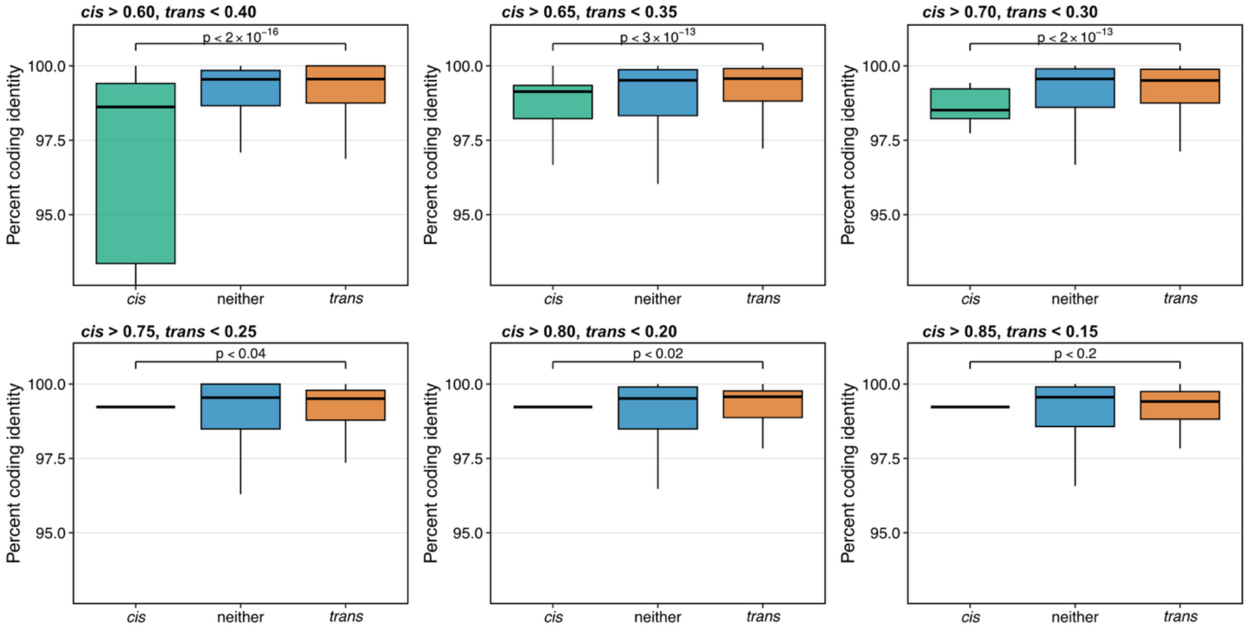

Figure S16. Percent coding identity (human-chimpanzee divergence) based on different mean *cis* proportion cutoffs of cell types DD to define *cis* and *trans* DD genes in individual cell types.

Supplementary Table S1 (provided as a separate file). Per gene estimates of dispersion and mean per sample (indicated by cell line ID and cell type annotation in the column names) from the cardiac HDC data as estimated by Memento. Dispersion estimates for permuted cell type annotations.

Supplementary Table S2 (provided as a separate file). Gene ontology enrichment. The first sheet contains enrichment results that were identified as low DD and high DE in each cell type comparison (indicated in the "contrasts" column). The second sheet contains enrichments for low-dispersion genes across cell types. The "cluster_resolution" column indicates at what resolution clustering was performed and dispersion was estimated to define low-dispersion genes. In both sheets, all other columns are standard outputs from clusterProfiler.

Supplementary Table S3 (provided as a separate file). All DE and DD results for both cardiac HDC and interspecies datasets (per gene effect sizes, standard errors, significance results). Detailed descriptions of each field provided in the file.

bioRxiv preprint doi: https://doi.org/10.64898/2026.04.01.715853; this version posted April 7, 2026. The copyright holder for this preprint (which was not certified by peer review) is the author/funder, who has granted bioRxiv a license to display the preprint in perpetuity. It is made available under aCC-BY 4.0 International license.

Cell Type	DD Genes	Recapitulated	% Recapitulated
Neuroblast	794	95	12.0%
ENS glia	787	51	6.5%
Radial glia	860	196	22.8%
Neuronal IPC	852	182	21.4%
Glioblast	833	81	9.7%

Supplementary Table S4. Number of genes per cell type in the diploid lines with significant species differences in dispersion (LFSR < 0.05). Number and percent of DD genes that are recapitulated (namely, regulated in *cis*) in the allotetraploid line.

Cell Type	*Cis*	Neither	*Trans*	Mixed
Neuroblast	91	316	387	-
ENS glia	103	333	351	-
Radial glia	53	160	647	-
Neuronal IPC	73	209	570	-
Glioblast	115	348	370	-
Overall	43	10	539	276
Overall mean-based	13	423	432	-

Supplementary Table S5. Number of DD genes that are classified as *cis*, *trans*, or neither (see Methods) per cell type. DD genes are classified as *cis* if the estimated cis proportion exceeds 0.7, as *trans* if it is below 0.3, and as *neither* otherwise. In the 'overall' analysis, which considered all cell type, 'mixed' refers to genes whose DD was accounted for by *cis* in at least one cell type and *trans* in at least one other cell type. The total number of genes per cell type (*cis* + *trans* + neither) equals the number of DD genes observed in the diploid lines ('DD genes' in Table S4).
